# Correction: *Wdr62* is involved in female meiotic initiation via activating JNK signaling and associated with POI in humans

**DOI:** 10.1371/journal.pgen.1008504

**Published:** 2019-11-15

**Authors:** Yang Zhou, Yan Qin, Yingying Qin, Binyang Xu, Ting Guo, Hanni Ke, Min Chen, Lianjun Zhang, Feng Han, Yaqiong Li, Min Chen, Axel Behrens, Yaqing Wang, Zhiheng Xu, Zi-Jiang Chen, Fei Gao

## Notice of republication

This article was republished on 10^th^ April 2019 to correct errors in the original article.

The authors originally reported that infertility in *Wdr62*-deficient mice was caused by defects in meiotic initiation, as presented and analyzed in embryonic ovaries and early (P3 and P5) postnatal testes. However, the original article did not include the finding that in older (21 days and later) male animals, meiotic initiation proceeds normally for second wave spermatogenesis, and that male germ cells in older mice exhibit metaphase I arrest. The mechanism of metaphase I arrest in older male germ cells, and the basis of the difference in *Wdr62* dependency in young and older male germ cells is currently under investigation. The article title and text have been amended in light of this finding.

Please download this article again to view the correct version. The originally published, uncorrected article and the republished, corrected article are provided here for reference.

## Supporting information

S1 File Originally published, uncorrected article. (PDF)

S2 File Republished, corrected article. (PDF)
