# Supplementary figures and images for: Wdr62 is involved in female meiotic initiation via activating JNK signaling and associated with POI in humans
Source: PLoS Genet. 2018 Aug 13;14(8):e1007463. doi: 10.1371/journal.pgen.1007463 (PMC6107287; doi:10.1371/journal.pgen.1007463)

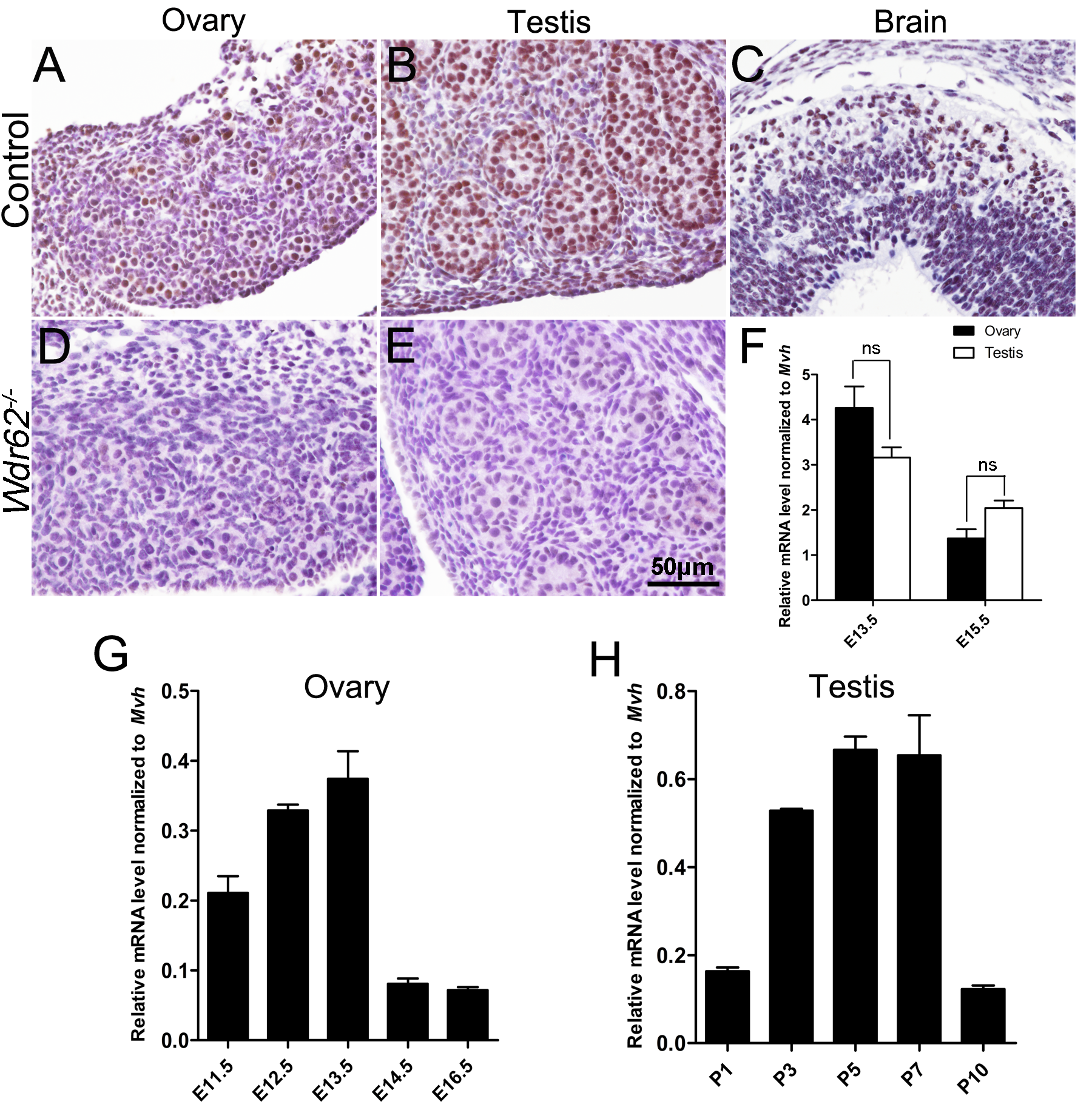

Supplement: S1 Fig — Wdr62 expression in the ovaries and testes was examined by immunohistochemistry and real-time PCR. WDR62 protein was abundantly expressed in (A) female and (B) male germ cells from control mice. No WDR62 protein was detected in germ cells from Wdr62-defecient (D) ovaries and (E) testes at E13.5. (C) Brain IHC staining as a positive control. (F) The mRNA level of Wdr62 in ovaries and testes at E13.5 and E15.5. (G) Wdr62 mRNA levels were gradually increased from E11.5 to E13.5 and dramatically decreased at E14.5 and E16.5 in female gonads. (H) In testes, Wdr62 mRNA levels were significantly increased from P1 to P7 and dramatically decreased at P10. Data are presented as the mean ± SEM. (TIF) [file pgen.1007463.s001.tif]

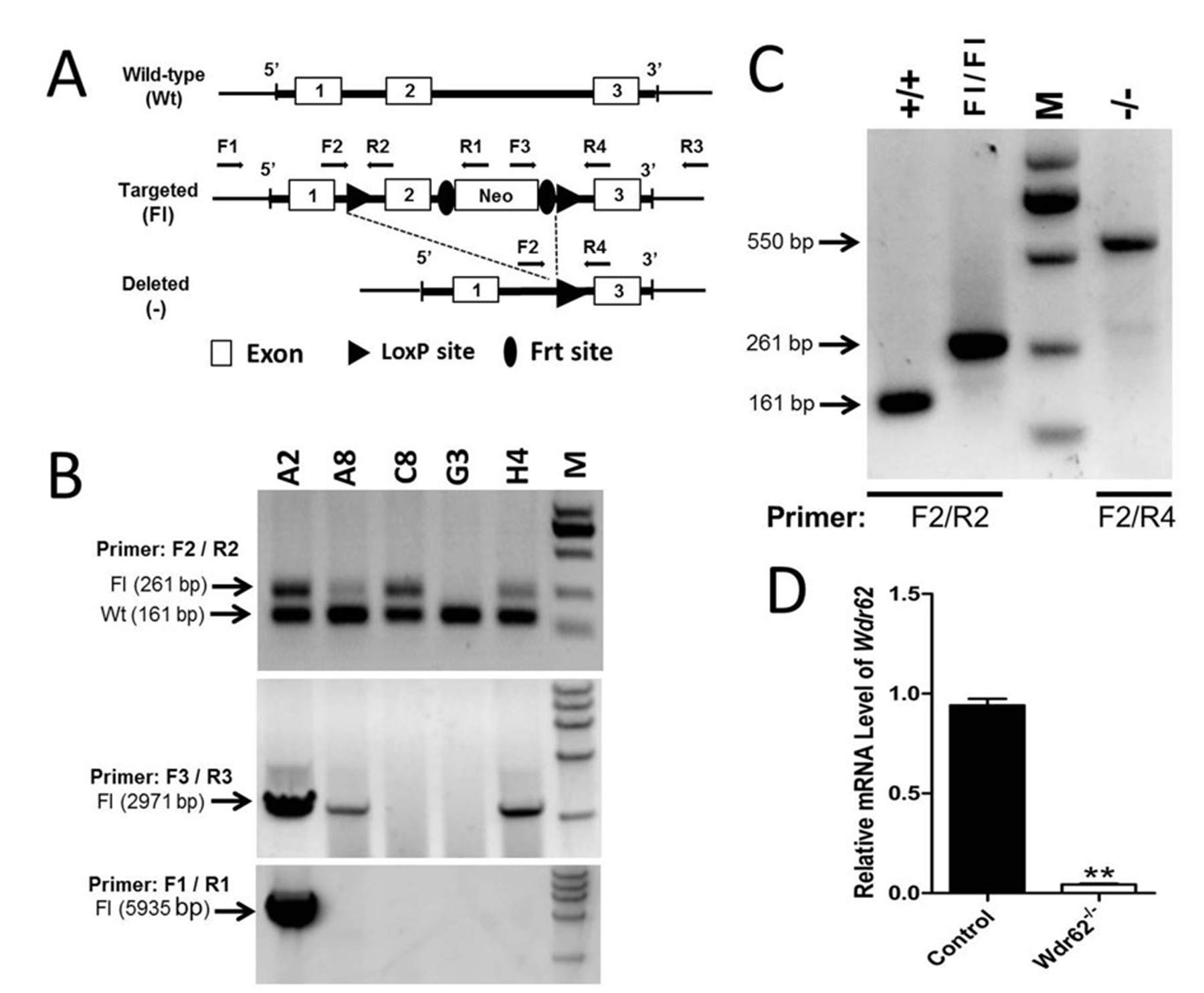

Supplement: S2 Fig — (A) Gene-targeting strategy for generating the Wdr62flox allele. (B) Upper panel: PCR analysis using primers F2/R2. Middle panel: PCR analysis using primers F3/R3. Lower panel: PCR analysis using primers F1/R1. ES clone A2 is the positive clone, containing both LoxP sites and Neo, and was homologously recombined into genomic DNA. (C) Genotyping of mice. +, WT; Fl, targeted; -, deleted. (D) Real-time PCR analysis showed the efficiency of Wdr62 knock-out in the female germ cells at E13.5. Data are presented as the mean ± SEM. ns, p > 0.05; *p < 0.05; **p < 0.01. (TIF) [file pgen.1007463.s002.tif]

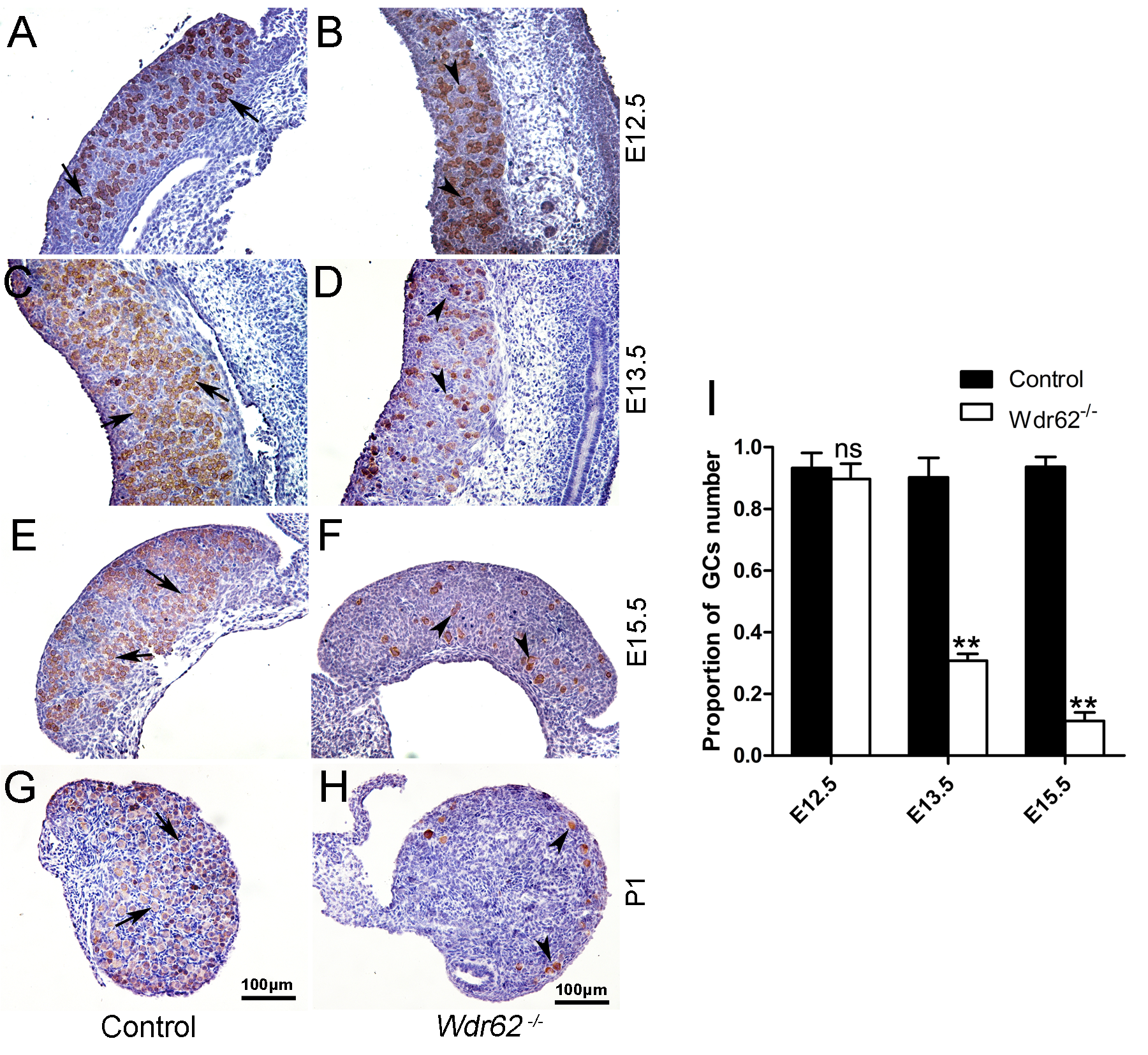

Supplement: S3 Fig — Germ cells were labeled with anti-MVH antibody. The number of germ cells in the (B) Wdr62−/− ovaries (black arrowheads) was not changed at E12.5 compared with (A) the control ovaries (black arrows). The number of MVH-positive germ cells was significantly reduced in Wdr62−/− ovaries at (D) E13.5 and (F) E15.5 compared with (C and E) control ovaries. (G) Numerous germ cells (black arrows) were observed in control ovaries at P1, whereas (H) very few MVH-positive germ cells (black arrowheads) were noted in Wdr62-deficient ovaries at P1. (I) Quantification of germ cell numbers in control and Wdr62−/− ovaries at different developmental stages. Data are presented as the mean ± SEM. ns, p > 0.05; *p < 0.05; **p < 0.01. (TIF) [file pgen.1007463.s003.tif]

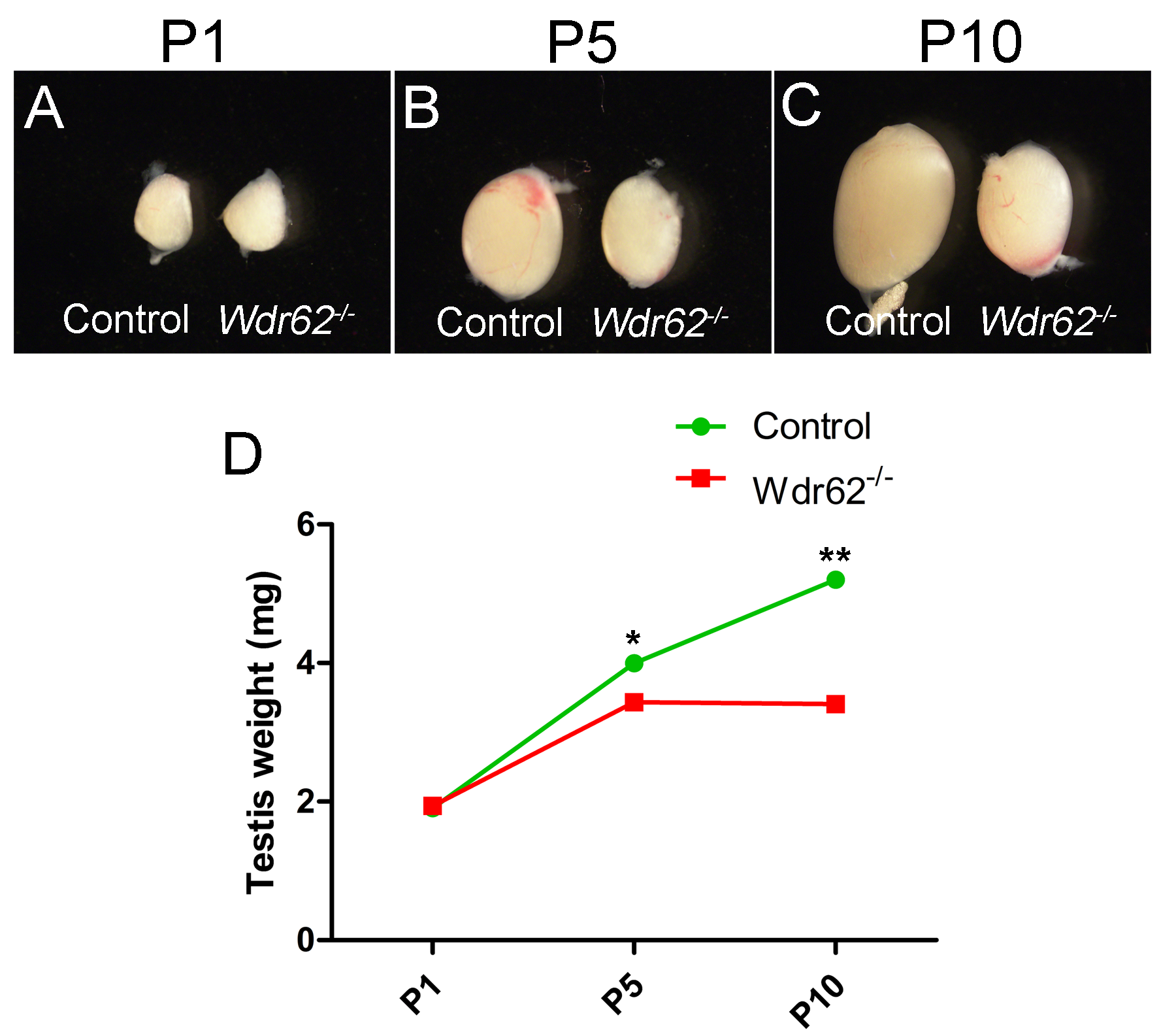

Supplement: S4 Fig — (A-C) The size of Wdr62−/− testes was comparable to the control testes at P1, and smaller than controls at P5 and P10. (D) The weight of Wdr62−/− testes was significantly decreased at P5 and P10. Data are presented as the mean ± SEM. ns, p > 0.05; *p < 0.05; **p < 0.01. (TIF) [file pgen.1007463.s004.tif]

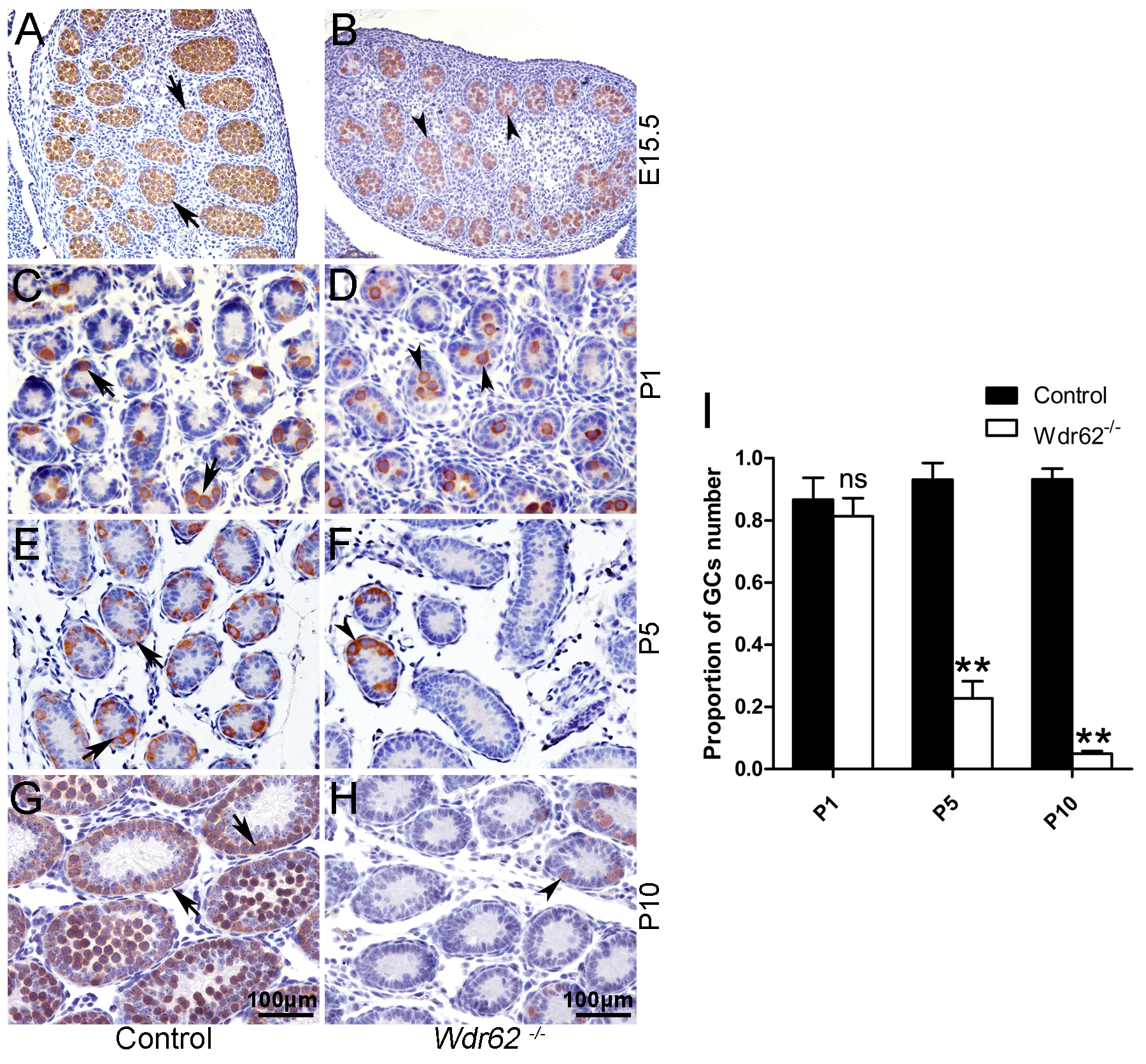

Supplement: S5 Fig — Germ cells were labeled with anti-MVH antibody. (B and D) The number of germ cells in Wdr62−/− testes was not changed at E15.5 and P1 (black arrowheads), respectively, compared with (A and C) the control testes (black arrows). (F) The germ cell loss in Wdr62−/− testes (black arrowheads) was noted at P5, and (H) very few germ cells were observed in Wdr62-deficient testes at P10 (black arrowheads). (I) Quantification of germ cell numbers in control and Wdr62−/− testes at different developmental stages. Data are presented as the mean ± SEM. ns, p > 0.05; *p < 0.05; **p < 0.01. (TIF) [file pgen.1007463.s005.tif]

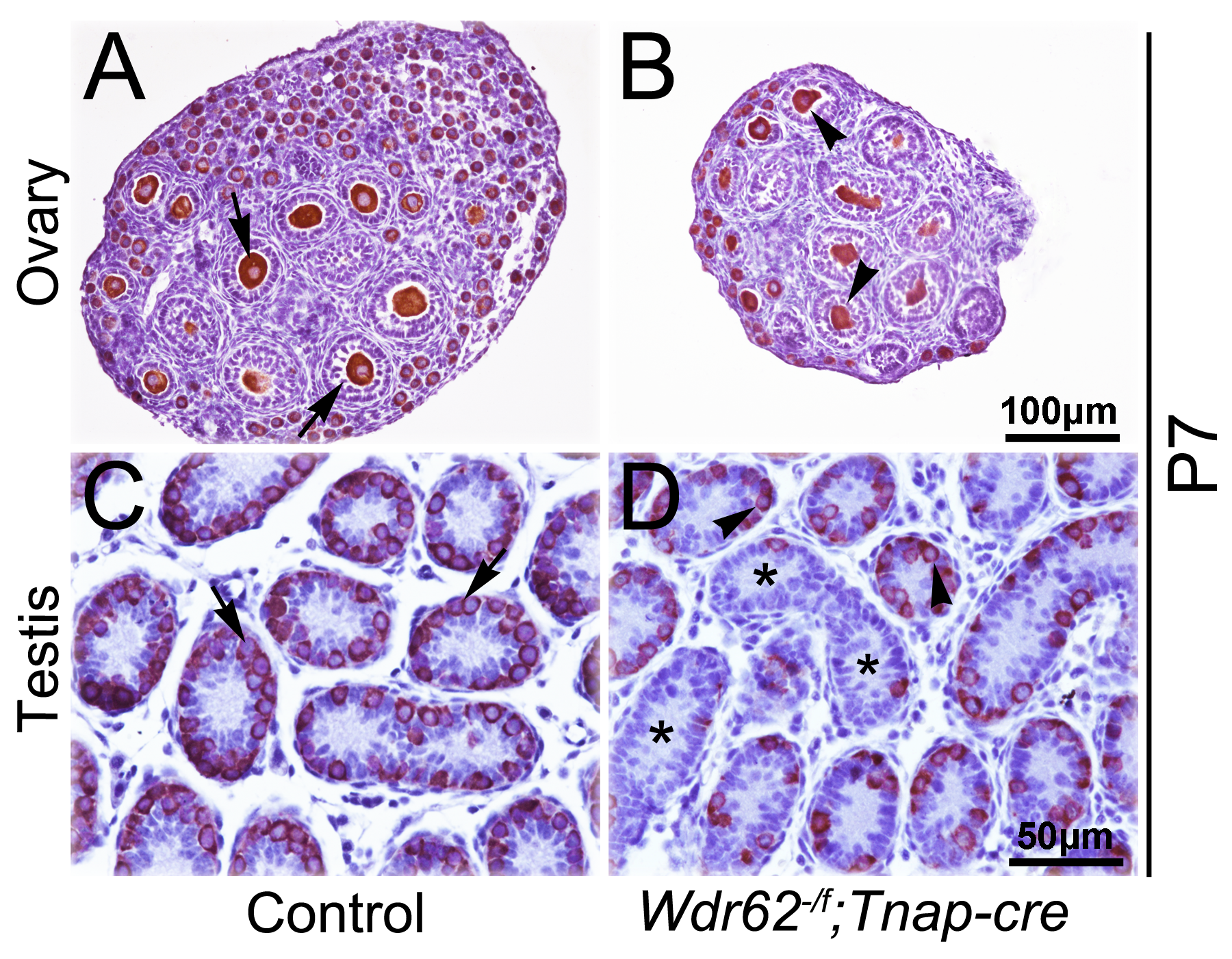

Supplement: S6 Fig — To examine the functions of Wdr62 was specifically in germ cells, Wdr62+/−; Tnap-Cre males were crossed with Wdr62flox/flox females to obtain Wdr62−/flox; Tnap-Cre offspring, in which Cre is activated in germ cells of ovaries and testes at approximately 8.5 dpc at embryo stage. It is shown that few germ cells were survived in the (B, black arrowheads) ovaries and (D, black arrowheads) testes of Wdr62−/flox; Tnap-Cre mice compared with that of (A and C, black arrows) control mice at P7. (TIF) [file pgen.1007463.s006.tif]

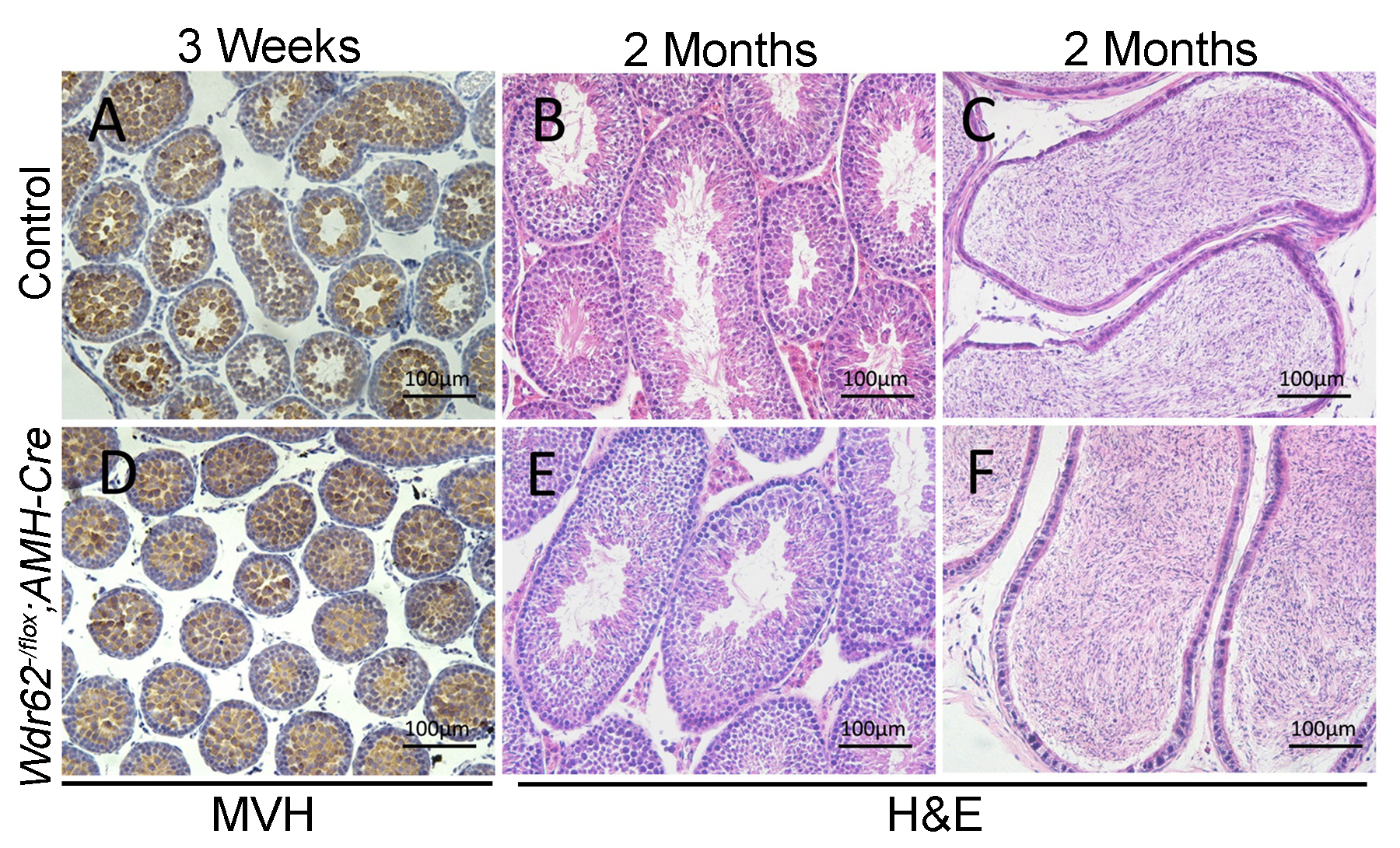

Supplement: S7 Fig — Compared with (A, B and C) control mice, the germ cell development in (D, E and F) Wdr62−/flox; Amh-Cre mice was not affected. (F) A large number of mature sperm were observed in the epididymis of Wdr62−/flox; Amh-Cre mice. (TIF) [file pgen.1007463.s007.tif]

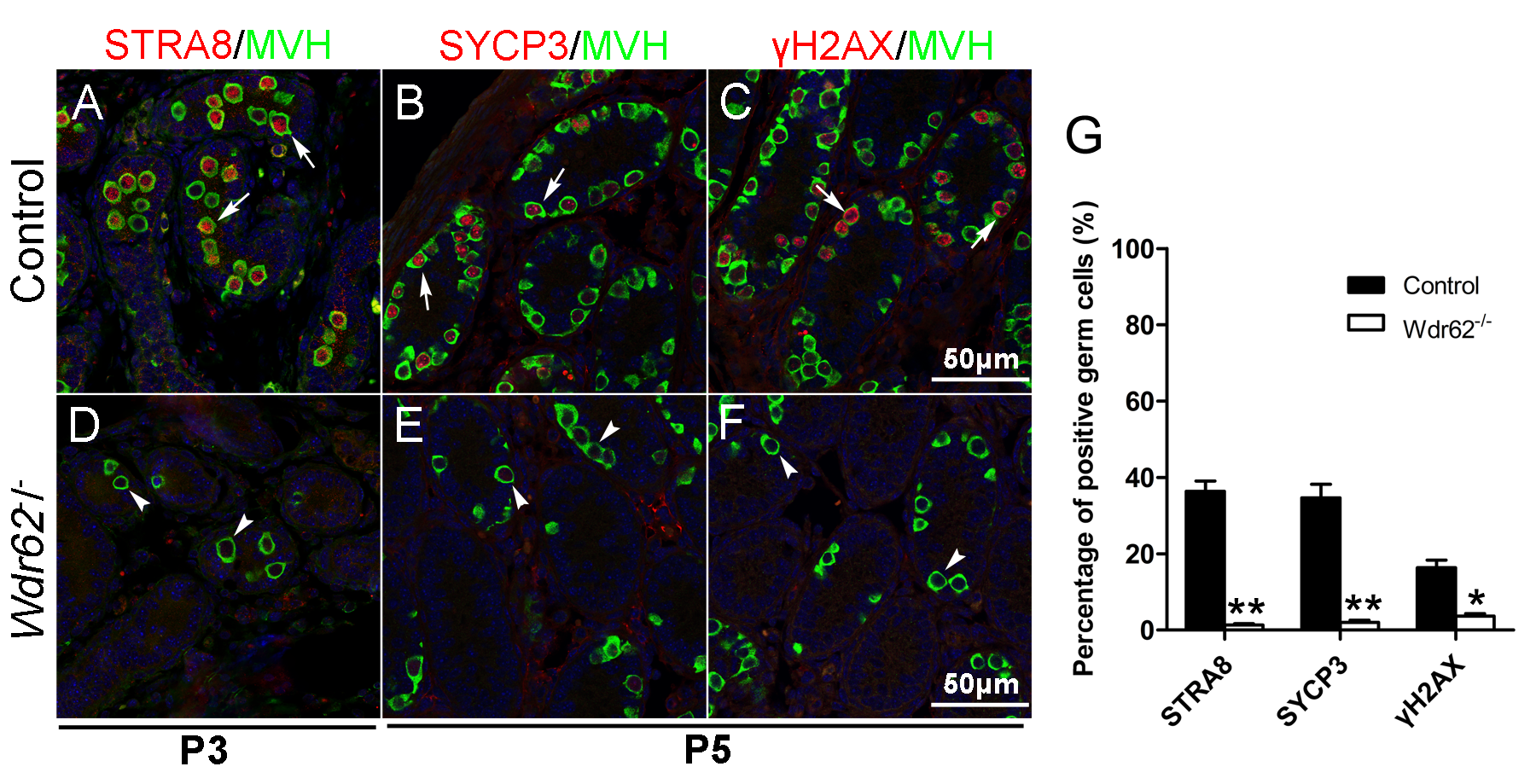

Supplement: S8 Fig — The expression of meiosis-related genes in germ cells was examined by immunofluorescence. In control testes, (A, red) STRA8 was detected in most germ cells (green, white arrows) at P3. (B, red, white arrows) SYCP3 and (C, red, white arrows) λH2AX were detected in control germ cells (green, white arrows) at P5. (D, E and F) None of these proteins was expressed in germ cells (white arrowheads) from Wdr62-deficient testes at these stages. (G) Quantitative analyses of meiotic germ cells from control and Wdr62-deficient testes at P3 and P5. Data are presented as the mean ± SEM. ns, p > 0.05; *p < 0.05; **p < 0.01. (TIF) [file pgen.1007463.s008.tif]

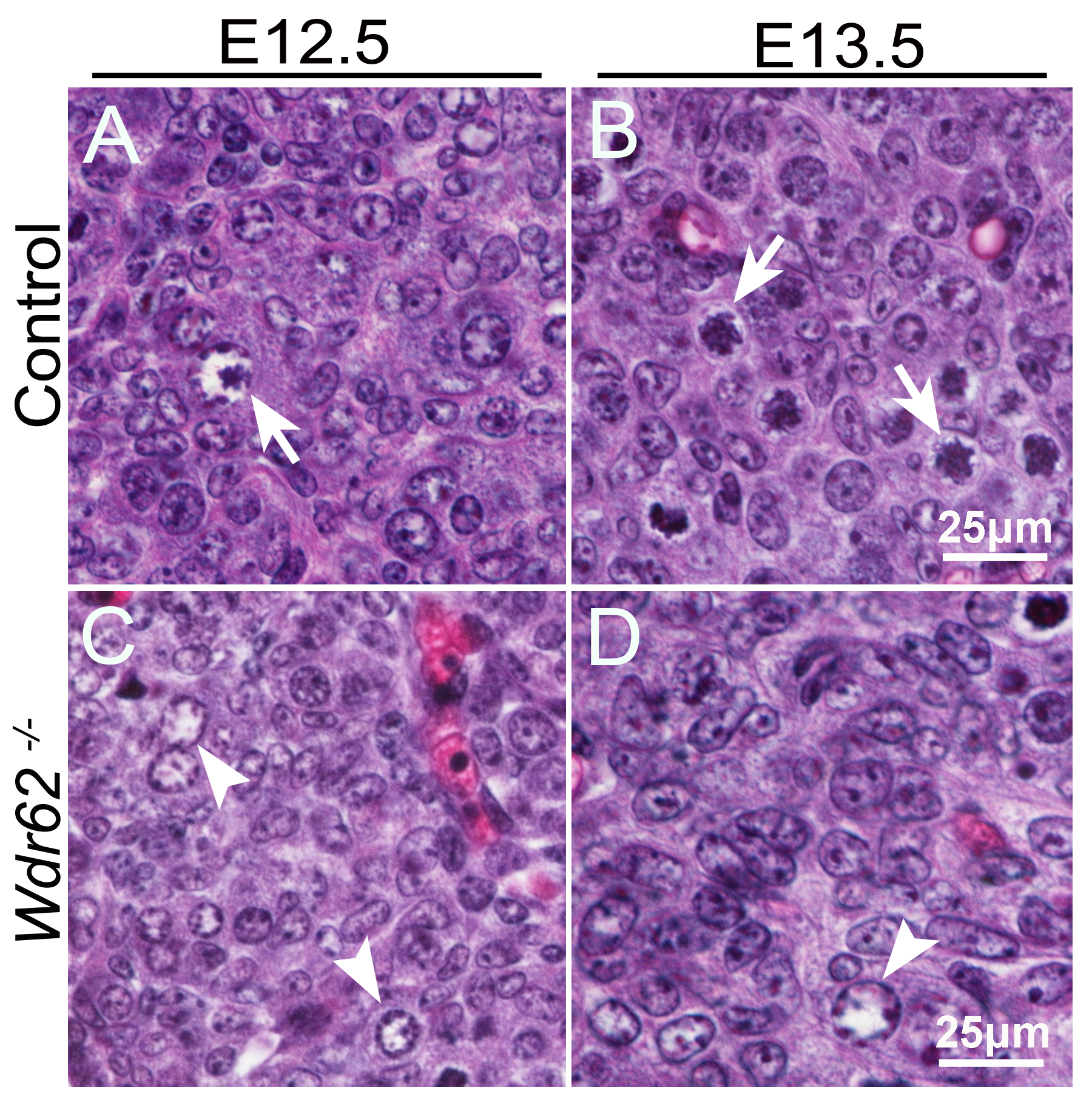

Supplement: S9 Fig — Hematoxylin and eosin staining of ovarian sections from the control and Wdr62-deficient littermate embryos. The germ cells displayed a morphology with patches of condensed chromatin at the periphery of the nucleus in both (A, white arrows) control and (C, white arrowheads) Wdr62−/− ovaries at E12.5. (B, white arrows) By E13.5, the nuclei in many control germ cells showed the thread–like chromosome condensation that represents the pre-leptotene stage, (D, white arrowheads) whereas the nuclei from Wdr62-deficient germ cells retained the morphology of the mitotic stage as observed at E12.5. (TIF) [file pgen.1007463.s009.tif]

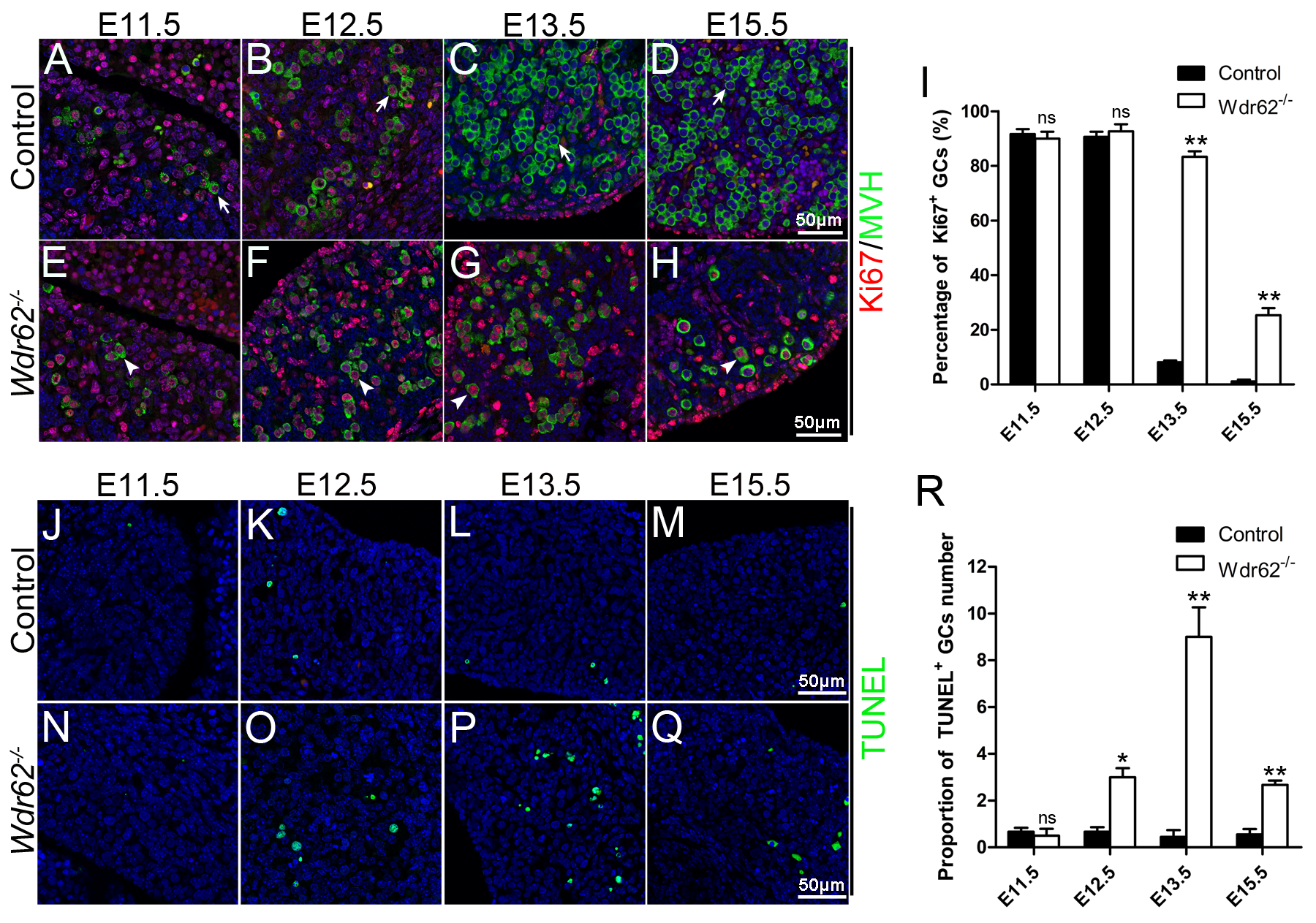

Supplement: S10 Fig — The proliferation and apoptosis of germ cells was examined by Ki67 staining and TUNEL assay. Ki67 signal (red) was detected in most germ cells (green, white arrows) at E11.5 and E12.5 from (A and B) control and (E and F) Wdr62-deficient ovaries. (C and D) Very few Ki67-positive germ cells were detected in control ovaries at E13.5 and E15.5, (G and H) whereas numerous germ cells in Wdr62-deficient ovaries were retained Ki67-positive at E13.5 and E15.5. (I) The quantification analyses of Ki67-positive germ cells in control and Wdr62−/− ovaries at different developmental stages. (J-Q) Representative images of TUNEL assay of control and Wdr62−/− ovaries. (R) Quantitative analyses of TUNEL-positive germ cells in control and Wdr62−/− ovaries. Data are presented as the mean ± SEM. ns, p > 0.05; *p < 0.05; **p < 0.01. (TIF) [file pgen.1007463.s010.tif]

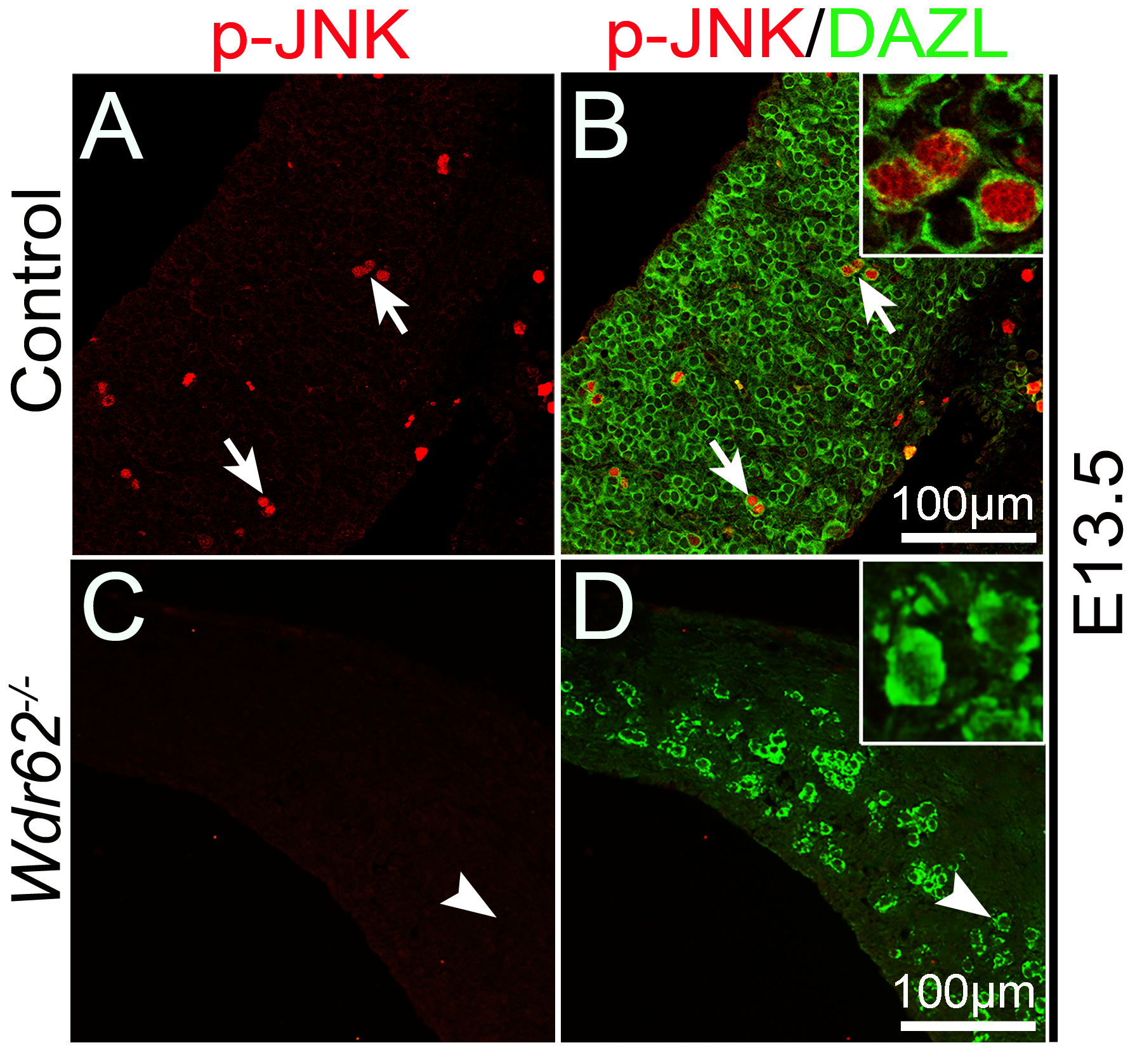

Supplement: S11 Fig — The expression of p-JNK in germ cells at E13.5 was examined by immunofluorescence. In (A and B) control mice, p-JNK was detected in a small portion of germ cells (green, white arrows), whereas very few p-JNK positive germ cell (green, white arrowheads) was noted in (C and D) Wdr62-deficient mice. (TIF) [file pgen.1007463.s011.tif]

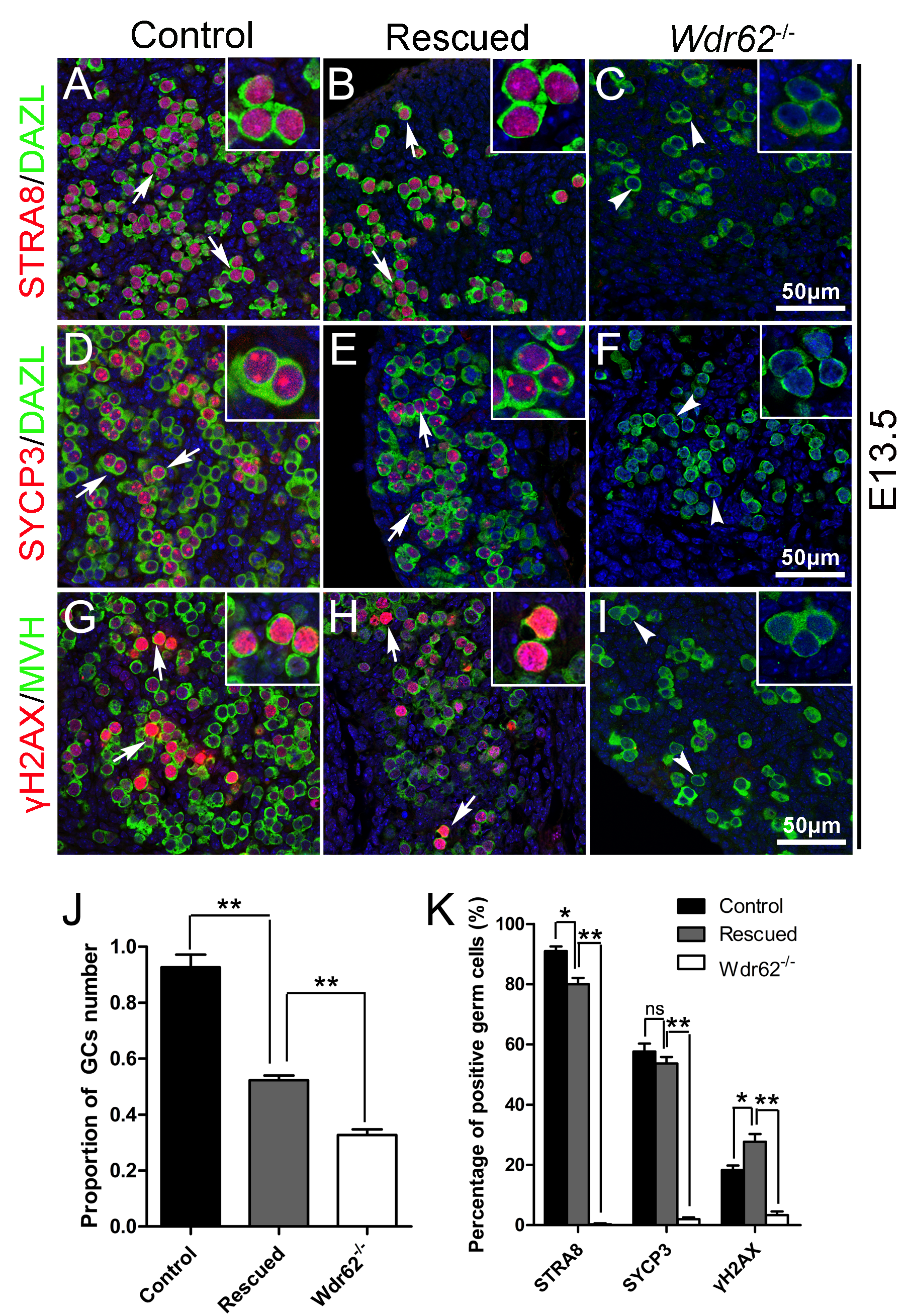

Supplement: S12 Fig — The expression of meiotic genes was examined by immunofluorescence. (A and B, white arrows) STRA8, (D and E, white arrows) SYCP3 and (G and H, white arrows) γH2AX were detected in germ cells from (A, D and G) control and (B, E and H) rescued ovaries, but not in the (C, F and I) Wdr62−/− ovaries. The number of germ cells was also significantly increased in the (B, E, and H) rescued ovaries compared with the (C, F and I) Wdr62−/− ovaries. (J) Quantification of germ cell numbers in control, rescued and Wdr62-deficient ovaries at E13.5. (K) Quantitative analyses of meiotic germ cells in control, rescued and Wdr62-deficient ovaries at E13.5. Data are presented as the mean ± SEM. ns, p > 0.05; *p < 0.05; **p < 0.01. (TIF) [file pgen.1007463.s012.tif]
