# Supplementary material for: Wdr62 is involved in female meiotic initiation via activating JNK signaling and associated with POI in humans
Source: PLoS Genet. 2018 Aug 13;14(8):e1007463. doi: 10.1371/journal.pgen.1007463 (PMC6107287; doi:10.1371/journal.pgen.1007463)
Supplement: S1 Table — (DOCX) [file pgen.1007463.s013.docx]

S1 Table. Primers used for real-time PCR and mutagenesis analysis.

| Gene Symbol | Forward Primer 5’ to 3’ | Reverse Primer 5’ to 3’ |
| --- | --- | --- |
| *Stra8* | CTGTTGCCGGACCTCATGG | TCACTTCATGTGCAGAGATGATG |
| *Rec8* | CTACCTAGCTTGCTTCTTCCCA | GCCTCTAAAAGGTGTCGAATCTG |
| *Sycp3* | AGAAATGTATACCAAAGCTTCTTTCAA | TTAGATAGTTTTTCTCCTTGTTCCTCA |
| *Dmc1* | CCCTCTGTGTGACAGCTCAAC | GGTCAGCAATGTCCCGAAG |
| *Spo11* | ATTCTGTCGGCCTTCGGATG | TTGCATAAGTGTCGCTCTGTATT |
| *Dazl* | ATGTCTGCCACAACTTCTGAG | CTGATTTCGGTTTCATCCATCCT |
| *Mvh* | AGGGGATGAAAGAACTATGGTC | AGCAACAAGAACTGGGCACT |
| *Oct4* | AGAGGATCACCTTGGGGTAC | CGAAGCGACAGATGGTGGTC |
| *Nanog* | AGGCTTTGGAGACAGTGAGGTG | TGGGTAAGGGTGTTCAAGCACT |
| *Sox2* | GCGGAGTGGAAACTTTTGTCC | CGGGAAGCGTGTACTTATCCTT |
| *Stella* | GACCCAATGAAGGACCCTGAA | GCTTGACACCGGGGTTTAG |
| *Msx1* | TCATGGCCGATCACAGGAAG | GGAGTCCTCCGACTGAGAAATG |
| *Msx2* | GGAGCACCGTGGATACAGG | TAGAAGCTGGGATGTGGTGAA |
| *Gapdh* | GTCATTGAGAGCAATGCCAG | GTGTTGCTACCCCCAATGTG |
| *Stra8* promoter | AAGCTTAAACTTGCCTCCAAGGGGGT | GGATCCCGACTGCCCGTCGCAGAATA |
| AP-1 promoter | CTGAGTCAGTGAGTCACTGACTCACTGACTCATGAGTCAGCTGACTCA GGAGCTCCACCGCGTATATAAGCA | GATCTGCTTATATACGCGGTGGAGCTCCTGAGTCAGCTGACTCATGAGTCAGTGAGTCAGTGACTCACTGACTCAGGTAC |
| WDR62-1 | ACCCACCTCACCACCTTTAG | GGTCCCTCAATTCCTTCCCA |
| WDR62-2 | TCTGCTGTGACTCATGGTGT | CTTGAACCAGGGAGTCGGAG |
| M1 | GACATCCAGATGATCAGCTATGGCGCCGACAAGAGCATC | GATGCTCTTGTCGGCGCCATAGCTGATCATCTGGATGTC |
| M2 | ACCACTTCGAGACACTGAGAGCCCCTGCAGAGAGCT | AGCTCTCTGCAGGGGCTCTCAGTGTCTCGAAGTGGT |
