# Supplementary material for: Wdr62 is involved in female meiotic initiation via activating JNK signaling and associated with POI in humans
Source: PLoS Genet. 2018 Aug 13;14(8):e1007463. doi: 10.1371/journal.pgen.1007463 (PMC6107287; doi:10.1371/journal.pgen.1007463)
Supplement: S2 Table — (DOCX) [file pgen.1007463.s014.docx]

S2 Table. Superovulation and embryo transplantation.

| Genotype | Number of embryos | Fertilization rate | Number of transferred embryos | Number of offspring |
| --- | --- | --- | --- | --- |
|  | 27 | 23/27 | 23 | 13 |
| Control | 30 | 28/30 | 28 | 15 |
|  | 23 | 21/23 | 21 | 11 |
|  | 6 | 6/6 | 6 | 1 |
| Rescued | 8 | 7/8 | 7 | 0 |
|  | 7 | 5/7 | 5 | 1 |
|  | 0 | 0/0 | 0 | 0 |
| *Wdr62^−/−^* | 1 | 0/1 | 0 | 0 |
|  | 2 | 0/2 | 0 | 0 |
