# Supplementary material for: Wdr62 is involved in female meiotic initiation via activating JNK signaling and associated with POI in humans
Source: PLoS Genet. 2018 Aug 13;14(8):e1007463. doi: 10.1371/journal.pgen.1007463 (PMC6107287; doi:10.1371/journal.pgen.1007463)
Supplement: S3 Table — (DOCX) [file pgen.1007463.s015.docx]

S3 Table. Clinical features of sporadic patients with PA and matched controls.

|  | PA | Control |
| --- | --- | --- |
| Number of cases | 50 | 192 |
| Age ^a^ | 27.44±4.28 | 27.82±4.79 |
| Age at menarche ^a^ | - | 14.8±2.7 |
| Basal FSH ^a^ (IU/L) | 71.56±25.98 | 6.39±1.56 |
| Basal LH ^a^ (IU/L) | 25.54±13.89 | 5.50±2.81 |
| Basal E2 ^b^ (IU/L) | 10.4(5.00~17.3) | 35.8(25.55~47.5) |

^a^ Data were expressed as mean±SD for continuous variables normally distributed.

^b^ Data were expressed as median (interquartile range) for continuous variables not normally distributed.
