# Supplementary material for: Correction: Wdr62 is involved in female meiotic initiation via activating JNK signaling and associated with POI in humans
Source: PLoS Genet. 2019 Nov 15;15(11):e1008504. doi: 10.1371/journal.pgen.1008504 (PMC6857852; doi:10.1371/journal.pgen.1008504)
Supplement: S1 File — (PDF) [file pgen.1008504.s001.pdf]

PLOS GENETICS

RESEARCH ARTICLE

Wdr62 is involved in meiotic initiation via activating JNK signaling and associated with POI in humans

Yang Zhou[1,2☯], Yan Qin[1,2☯], Yingying Qin[3☯], Binyang Xu[1,2], Ting Guo[3], Hanni Ke[3], Min Chen[1], Lianjun Zhang[1,2], Feng Han[1,2], Yaqiong Li[1,2], Min Chen[1,2], Axel Behrens[4], Yaqing Wang[5], Zhiheng Xu[5]*, Zi-Jiang Chen[3]*, Fei Gao[1,2]*

1 State Key Laboratory of Reproductive Biology, Institute of Zoology, Chinese Academy of Sciences, Beijing, China, **2** University of Chinese Academy of Sciences, Beijing, China, **3** Center for Reproductive Medicine of Shandong University, National Research Center for Assisted Reproductive Technology and Reproductive Genetics, The Key Laboratory for Reproductive Endocrinology of Ministry of Education, Jinan, China, **4** CR-UK London Research Institute, London, United Kingdom, **5** State Key Laboratory of Molecular Developmental Biology, Institute of Genetics and Developmental Biology, Chinese Academy of Sciences, Beijing, China

☯ These authors contributed equally to this work.
* zhihengxu1966@163.com (ZX); chenzijiang@hotmail.com (ZJC); gaof@ioz.ac.cn (FG)

Check for updates

OPEN ACCESS

Citation: Zhou Y, Qin Y, Qin Y, Xu B, Guo T, Ke H, et al. (2018) *Wdr62* is involved in meiotic initiation via activating JNK signaling and associated with POI in humans. PLoS Genet 14(8): e1007463. https://doi.org/10.1371/journal.pgen.1007463

Editor: Josephine Bowles, University of Queensland, UNITED STATES

Received: December 21, 2017

Accepted: June 1, 2018

Published: August 13, 2018

Copyright: © 2018 Zhou et al. This is an open access article distributed under the terms of the Creative Commons Attribution License, which permits unrestricted use, distribution, and reproduction in any medium, provided the original author and source are credited.

Data Availability Statement: All relevant data are within the paper and its Supporting Information files.

Funding: This work was supported by National key R&D program of China (2016YFA0500901); The National Science Fund for Distinguished Young Scholars (81525011); Strategic Priority Research Program of the Chinese Academy of Sciences (XDB19000000); The National Natural Science Foundation of China (31471348, 31601193, and 31671496). The funders had no role in study

Abstract

Meiosis is a germ cell-specific division that is indispensable for the generation of haploid gametes. However, the regulatory mechanisms of meiotic initiation remain elusive. Here, we report that the *Wdr62* (*WD40-repeat protein 62*) is involved in meiotic initiation as a permissive factor rather than an instructive factor. Knock-out of this gene in a mouse model resulted in meiotic initiation defects. Further studies demonstrated that *Wdr62* is required for RA-induced *Stra8* expression via the activation of JNK signaling, and the defects in meiotic initiation from *Wdr62*-deficient mice could be partially rescued by JNK1 overexpression in germ cells. More importantly, two novel mutations of the *WDR62* gene were detected in patients with premature ovarian insufficiency (POI), and these mutations played dominant-negative roles in regulating *Stra8* expression. Hence, this study revealed that *Wdr62* is involved in meiotic initiation via activating JNK signaling, which displays a novel mechanism for regulating meiotic initiation, and mutation of *WDR62* is one of the potential etiologies of POI in humans.

Author summary

Meiosis is a unique cell division process which is indispensable for the generation of haploid gametes. However, the regulatory mechanism of meiotic initiation is unclear. In this study, we demonstrated that *Wdr62* is required for meiotic initiation in germ cells via activating JNK signaling. More importantly, we also found that mutation of *WDR62* was a potential etiologies of POI in humans. Taken together, this study revealed a novel mechanism for regulating meiotic initiation, and a potential etiologies of POI in humans. The

design, data collection and analysis, decision to publish, or preparation of the manuscript.

Competing interests: The authors have declared that no competing interests exist.

results of this study provide important information for better understanding the regulation of meiotic initiation.

Introduction

In mammals, the haploid gametes are generated via meiosis, a program of two successive cell divisions preceded by one round of DNA replication. The onset of this program is referred to as meiotic initiation. Several intrinsic and extrinsic factors have been demonstrated to play roles in meiotic initiation. Retinoic acid (RA), the active derivative of vitamin A, which is synthesized in the mesonephros and diffuses into the adjacent gonad, is one of the most important meiosis-inducing factors [1, 2]. As an extrinsic factor, RA induces the germ cells to express the gatekeeper gene of meiosis *Stra8* (*stimulated by retinoic acid gene 8*) [3]. Although the molecular functions of *Stra8* have not yet been identified, several studies have shown that it is the first detectable sign of a germ cell's decision to enter meiosis and is essential for pre-meiotic DNA replication and subsequent meiotic initiation [4±6]. Additionally, the RNA-binding protein DAZL (deleted in azoospermia-like) is an intrinsic factor required for germ cells to initiate the process of meiosis. *Dazl* knock-out mice fail to express meiotic marker genes in germ cells and retain a PGC (primordial germ cells) -like state in both sexes [7, 8]. Although the morphological changes during meiosis have been extensively studied, the underlying mechanisms that initiate this process remain largely unknown.

Premature ovarian insufficiency (POI), which is characterized by menstrual disturbance (oligomenorrhea or amenorrhea), elevated gonadotropins and low estradiol before 40 years of age, affects approximately 1% of women of childbearing age [9]. POI is heterogeneous in etiology, and known causes include genetic, autoimmune, iatrogenic or idiopathic factors. Approximately 25% of cases are thought to be genetically associated, with mutations in more than 80 genes concerning gonadal development, DNA replication/meiosis, DNA repair, and hormone synthesis [10±15]. However, up to date, etiology in most patients remains unknown.

WDR62 was originally identified as a scaffold protein in the JNK signaling pathway. *Wdr62* encodes a protein containing 13 WD40 domain repeats in its N-terminal half and MKK7/JNK binding domains and six potential JNK phosphorylation sites in its C-terminal half [16]. WDR62 is the second most common genetic alterations associated with microcephaly (MCPH) in humans [17]. The requirement of *Wdr62* in brain development and neural stem cell expansion has also been confirmed in mouse models [18±20].

Strikingly, we found that inactivation of *Wdr62* caused meiotic initiation defects and germ cell loss in this study. Further studies revealed that the meiotic defects in *Wdr62*-deficient mice could be partially rescued by JNK1 overexpression. Interestingly, two novel *WDR62* mutations were detected in 2 sporadic cases with POI, suggesting that mutation of *WDR62* is one of the potential etiologies of POI in humans.

Results

Inactivation of *Wdr62* caused germ cell loss in both females and males

The immunohistochemistry results showed that the WDR62 protein was abundantly expressed in the germ cells in both ovaries and testes during the embryonic stage (S1A and S1B Fig). The results of real time PCR showed that the mRNA level of *Wdr62* had no significant difference between ovaries and testes at E13.5 and E15.5 (S1F Fig). *Wdr62* mRNA levels gradually increased from E11.5 to E13.5, and dramatically decreased at E14.5 and E16.5 in female gonads

(S1G Fig). In testes, *Wdr62* expression significantly increased from P1 to P7 and dramatically decreased at P10 (S1H Fig). A knock-out mouse model was generated to investigate the function of *Wdr62* in germ cell development (S2 Fig). *Wdr62*$^{-/-}$ mice were born with a normal Mendelian ratio, and no developmental defects were observed (Fig 1A). When male and female *Wdr62*$^{-/-}$ mice were crossed with wild-type mice, no pups were obtained within 6 months, indicating that *Wdr62*$^{-/-}$ mice were completely infertile (Fig 1C and 1D). The size of ovaries in 2-month-old *Wdr62*-deficient females (Fig 1B, right) was dramatically reduced compared with that of control littermates (Fig 1B, left). H&E staining results showed that the ovarian follicles were absent in the *Wdr62*-deficient mice (Fig 1F). Further study found that the number of germ cells in *Wdr62*-deficient ovaries (S3B Fig) was comparable to that in control ovaries (S3A Fig) at E12.5. Germ cell loss was first noted in the *Wdr62*-deficient ovaries at E13.5 (S3D Fig). Germ cell number was dramatically decreased in the *Wdr62*-deficient ovaries at E15.5 (S3F Fig), and only a few MVH-positive germ cells remained at P1 (S3H Fig). The results of quantitative analyses also showed that the germ cell number was significantly reduced in *Wdr62*$^{-/-}$ ovaries after E13.5 (S3I Fig). As shown in S4 Fig, the size and weight of testes in *Wdr62*-deficient males were comparable to control littermates at P1. The size and weight of *Wdr62*$^{-/-}$ testes were slightly reduced at P5 and dramatically reduced at P10. The development of germ cells in *Wdr62*$^{-/-}$ testes (S5B and S5D Fig) was not affected at E15.5 and P1. The germ cell number was significantly reduced at P5 (S5F and S5I Fig), and very few of them were observed at P10 (S5H and S5I Fig).

Further confirm that *Wdr62* is involved in germ cell development with a cell autonomous function, *Wdr62*$^{-/flox}$*; Tnap-Cre* mice were generated. In *Tnap-Cre* mice, Cre recombinase is specifically expressed in germ cells of both male and female gonads at approximately E8.5 [21]. We found that the germ cell number was dramatically reduced in both male and female gonads 7 days after birth (S6 Fig). We also noticed that the phenotype observed in *Wdr62*$^{-/flox}$*; Tnap-Cre* mice was less severe than that in *Wdr62*$^{-/-}$ mice. This is most likely due to the relative low efficiency of *Tnap-Cre* activity. The leakage of *Tnap-Cre* is another issue which needs to be clarified. For this reason, we specifically deleted *Wdr62* in Sertoli cells using *AMH-Cre* mice. As shown in the S7 Fig, germ cell development in *Wdr62*$^{-/\mathrm{flox}}$*; Amh-Cre* mice was not affected, a large number of mature sperm were observed in the epididymis and histology of testes is normal. These results indicated that the defect of germ cell development was directly caused by inactivation of *Wdr62* in germ cells rather than in somatic cells.

Inactivation of *Wdr62* caused defect in meiotic initiation in germ cells

The timing of germ cell loss in *Wdr62*-deficient mice was consistent with the developmental stage for meiotic initiation. To examine whether the germ cell loss in *Wdr62*-deficient mice was caused by the defect in meiotic initiation, the expression of meiosis-specific marker genes was analyzed by immunofluorescence and real-time PCR. STRA8 protein was detected in the germ cells of control ovaries at E12.5 (Fig 2A) and E13.5 (Fig 2B), but not in germ cells from *Wdr62*$^{-/-}$ ovaries (Fig 2E and 2F). SYCP3 (Fig 2C) and γH2AX (Fig 2D) proteins were detected in most of the germ cells in control ovaries at E13.5, but these proteins were virtually absent in germ cells from *Wdr62*$^{-/-}$ ovaries (Fig 2G and 2H). However, germ cell marker proteins, DAZL and MVH, were expressed in both control (Fig 2A±2D) and *Wdr62*-deficient ovaries (Fig 2E±2H) at E12.5 and E13.5. The results of quantitative analyses showed that the percentage of STRA8-, SYCP3- and γH2AX-positive germ cells was significantly decreased in *Wdr62*$^{-/-}$ ovaries compared with control ovaries at E13.5 (Fig 2I). As shown in Fig 2J, the mRNA levels of meiotic genes were all significantly reduced in the purified *Wdr62*$^{-/-}$ germ cells at E13.5, whereas the expression of the germ cell-specific genes *Dazl* and *Mvh* was not

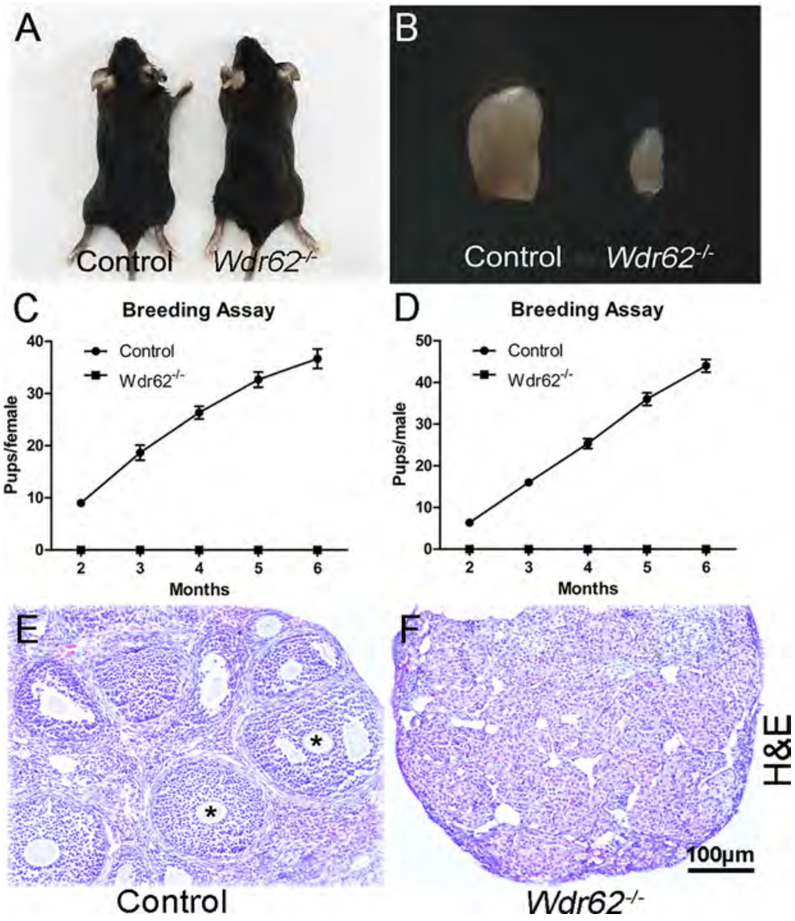

Fig 1. Inactivation of *Wdr62* causes infertility of both male and female mice. (A) *Wdr62*$^{-/-}$ mice were viable and no obvious developmental abnormities were noted at adult stage. (B) The size of ovary from *Wdr62*$^{-/-}$ mice was dramatically reduced compared with that of control mice. (C and D) Both female and male *Wdr62*$^{-/-}$ mice were completely infertile. The ovarian follicles at different developmental stages were observed in (E, asterisks) control ovaries at 2 month of age, but not in ovaries from (F) *Wdr62*$^{-/-}$ mice. Data are presented as the mean ± SEM. ns, $p > 0.05$, $^{*}p < 0.05$; and $^{**}p < 0.01$.

https://doi.org/10.1371/journal.pgen.1007463.g001

changed. Interestingly, the mRNA levels of pluripotency genes *Oct4*, *Nanog*, *Sox2* and *Stella* were significantly increased in the purified *Wdr62*-deficient germ cells, indicating that *Wdr62*-deficient germ cells were retained in an undifferentiated state.

We also examined the expression of meiotic genes in male germ cells. In control testes, STRA8 protein (S8A Fig) was expressed in most of germ cells at P3, and SYCP3 (S8B Fig) and λH2AX (S8C Fig) proteins were detected in the germ cells at P5. By contrast, none of these proteins were observed in germ cells from *Wdr62*-deficient testes at these stages (S8D, S8E and S8F Fig). The percentage of STRA8-, SYCP3- and γH2AX-positive germ cells was dramatically decreased in *Wdr62*$^{-/-}$ testes compared with control testes (S8G Fig). The H&E staining results showed that the germ cells displayed patches of condensed chromatin at the periphery of the nucleus in both control and *Wdr62*$^{-/-}$ ovaries at E12.5 (S9A and S9C Fig). By E13.5, the nuclei in control germ cells showed thread-like chromosome condensation that represents preleptotene, an initial stage of meiotic prophase (S9B Fig), whereas the nuclei from *Wdr62*-deficient germ cells still retained the same morphology as at E12.5 (S9D Fig). All these results indicated that *Wdr62* knockout caused a defect in meiotic initiation in germ cells.

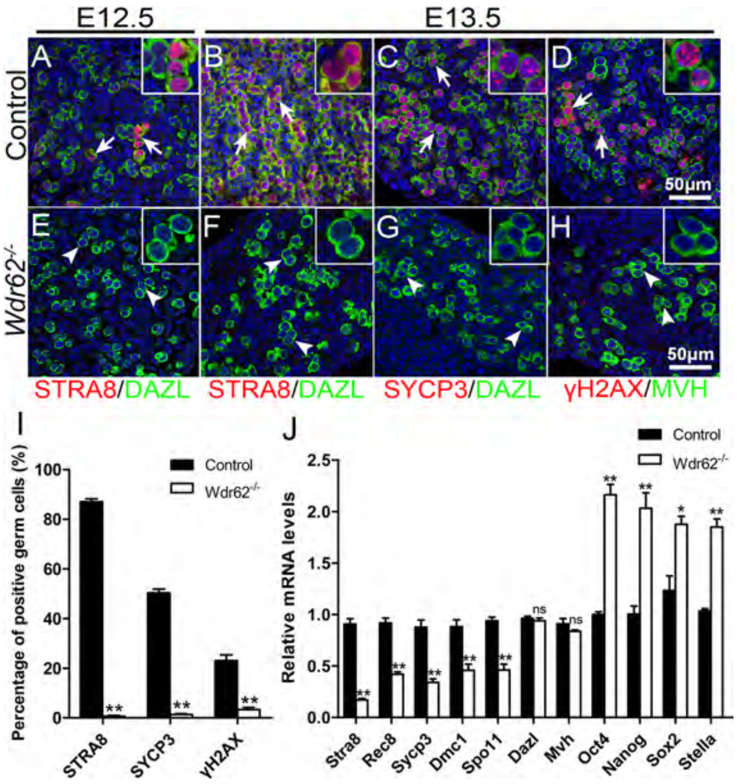

Fig 2. The expression of meiosis-related genes was dramatically reduced in germ cells from *Wdr62*$^{-/-}$ ovaries. The expression of meiosis-related genes in germ cells from control and *Wdr62*-deficient ovaries was examined by immunofluorescence and real-time PCR analysis. In control ovaries, STRA8 protein (red) was detected in a few germ cells (green, white arrows) at (A) E12.5 and in most of germ cells at (B) E13.5. No STRA8 protein was expressed in germ cells (green) from *Wdr62*$^{-/-}$ ovaries at (E) E12.5 and (F) E13.5. (C, red) SYCP3 and (D, red) γH2AX proteins were detected in most of germ cells (green, white arrows) in control ovaries at E13.5. No (G) SYCP3 or (H) γH2AX proteins were detected in germ cells (green, white arrowheads) from *Wdr62*$^{-/-}$ ovaries at E13.5. (I) Quantitative analyses of meiotic germ cells in control and *Wdr62*$^{-/-}$ ovaries at E13.5. (J) The expression of meiotic genes was analyzed by RT-PCR using purified germ cells. The mRNA levels of meiotic genes were also significantly reduced in purified *Wdr62*$^{-/-}$ germ cells at E13.5, whereas the expression of *Dazl* and *Mvh* was not changed. The expression of the pluripotent genes *Oct4*, *Nanog*, *Sox2* and *Stella* was dramatically increased in purified *Wdr62*-deficient germ cells. Data are presented as the mean ± SEM. ns, $p > 0.05$, $^*p < 0.05$; and $^{**}p < 0.01$.

https://doi.org/10.1371/journal.pgen.1007463.g002

To examine whether the germ cell loss in *Wdr62*-deficient ovaries is due to the defective proliferation or cell apoptosis, Ki67 immunostaining and TUNEL assay were performed. As shown in S10 Fig, a majority of germ cells were Ki67-positive in control and *Wdr62*-deficient ovaries at E11.5 and E12.5. Numerous germ cells were still Ki67-positive in *Wdr62*-deficient ovaries at E13.5 and E15.5. By contrast, most of germ cells in control ovaries were Ki67-negative at E13.5 and E15.5. The results of TUNEL assay showed that the number of apoptotic germ cells was slightly increased at E12.5 and dramatically increased at E13.5 in *Wdr62*-deficient ovaries. These results indicated that the loss of germ cell in *Wdr62*-deficient ovaries is not due to the defect of proliferation. The *Wdr62*-deficient germ cells were retained in an undifferentiated state and underwent apoptosis eventually.

Wdr62 is required for RA-induced meiotic gene expression as an intrinsic factor in germ cells

In male gonads, meiotic genes are not expressed in the germ cells during the embryonic stage, but can be induced by exogenous RA treatment [1, 2]. To test whether *Wdr62* is required for

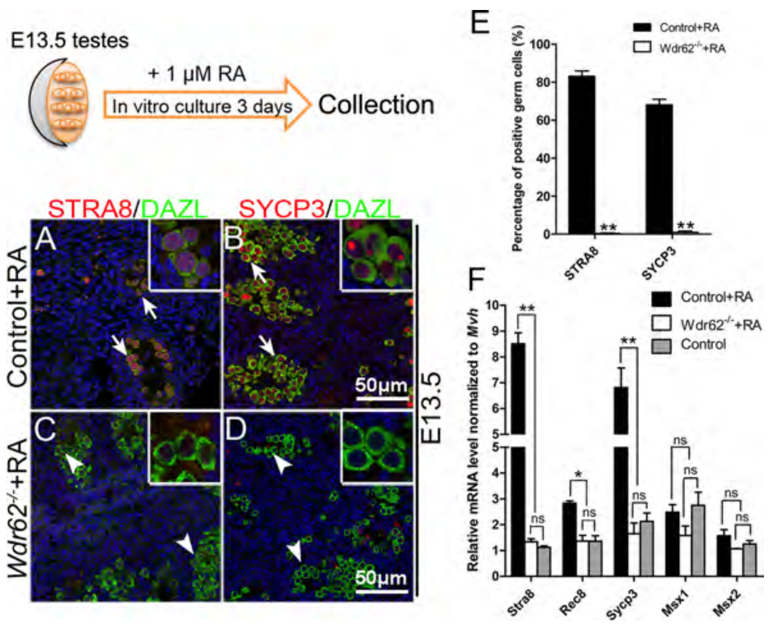

Fig 3. *Stra8* and *Sycp3* expression was not induced by RA treatment in germ cells from *Wdr62*$^{-/-}$ testes during the embryonic stage. The testes from E13.5 control and *Wdr62*$^{-/-}$ embryos were cultured in vitro for 72 hours and treated with 1 μM RA. The expression of meiotic genes was examined by immunofluorescence and real-time PCR analysis. Both (A, red) STRA8 and (B, red) SYCP3 proteins were detected in germ cells (green, white arrows) from control testes. No (C) STRA8 or (D) SYCP3 proteins were detected in germ cells (green, white arrowheads) from *Wdr62*$^{-/-}$ testes. (E) Quantitative analyses of STRA8- and SYCP3-positive germ cells in RA-treated control and *Wdr62*$^{-/-}$ testes in vitro at E13.5. (F) The mRNA levels of meiotic genes were significantly increased in the control testes with RA treatment, whereas it was not induced by RA treatment in the *Wdr62*$^{-/-}$ testes. Data are presented as the mean ± SEM. ns, $p > 0.05$, $^{*}p < 0.05$; and $^{**}p < 0.01$.

https://doi.org/10.1371/journal.pgen.1007463.g003

RA-induced meiotic genes expression, the testes from control and *Wdr62*$^{-/-}$ mice were dissected at E13.5 and cultured in the presence of 1 μM RA. As shown in Fig 3A±3D, STRA8 and SYCP3 proteins were detected in the germ cells from control testes, whereas no STRA8 and SYCP3 signals were noted in germ cells from *Wdr62*-deficient testes. The results of quantitative analyses showed that the percentage of STRA8- and SYCP3-positive germ cells was significantly reduced in *Wdr62*-deficient testes (Fig 3E). The mRNA levels of *Stra8*, *Sycp3* and other meiotic genes were also dramatically increased in the control testes after RA treatment but not in the *Wdr62*-deficient testes (Fig 3F). An in vitro study also showed that *Stra8* mRNA level was significantly induced by *Wdr62* in F9 cells in the presence of RA, but could not be induced by only *Wdr62* transfection, indicating that *Wdr62* seems to be a permissive factor but not an instructive factor (Fig 4A). Further study revealed that the *Stra8* promoter could not be activated by either the WD40 domain or the JNK binding domain of WDR62 (Fig 4B), indicating that both WD40 and MKK7/JNK binding domains are essential for the normal WDR62 function. All these results indicated that *Wdr62* is required for RA-induced *Stra8* expression as a permissive factor.

Wdr62 is involved in RA-induced meiotic gene expression by activating JNK signaling

It has been previously demonstrated that JNK signaling is activated by *Wdr62* [16, 18, 22]. It is reasonable to postulate that JNK signaling pathway may also be involved in *Wdr62*-dependent *Stra8* expression. The phosphorylated JNK protein in germ cells at E13.5 was examined by

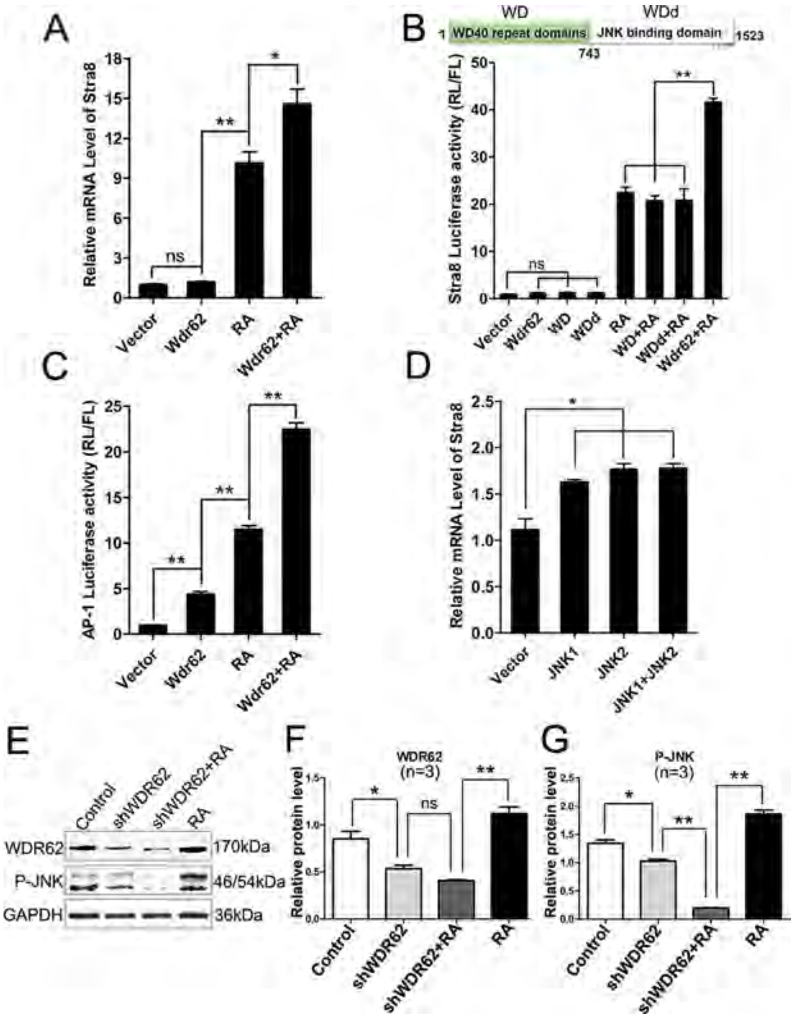

Fig 4. The *Stra8* promoter was activated by *Wdr62* and JNK signaling. (A) *Stra8* expression was significantly induced by *Wdr62* when retinoic acid (RA) was present. (B) *Stra8* promoter activity could not be activated by either the WDR62 protein WD40 or JNK binding domain combined with RA treatment. (C) The AP-1 promoter was synergistically activated by *Wdr62* transfection and RA treatment. (D) *Stra8* mRNA levels were significantly increased in F9 cells when JNK1 and/or JNK2 was overexpressed. (E) *Wdr62* was required for RA-induced JNK signaling activation. (F-G) Relative protein levels of WDR62 and P-JNK. Data are presented as the mean ± SEM (n = 3). ns, $p > 0.05$, $^{*}p < 0.05$; and $^{**}p < 0.01$.

https://doi.org/10.1371/journal.pgen.1007463.g004

immunostaining. We found that p-JNK was detected in a small portion of germ cells in control mice (S11A and S11B Fig). By contrast, very few p-JNK positive germ cell was noted in *Wdr62*-deficient mice (S11C and S11D Fig). These results suggest that the activation of JNK signaling is probably involved in germ cell meiotic initiation. Moreover, a luciferase assay with AP-1 promoter, which is a direct downstream factor of JNK signaling, was performed. As expected, the activity of the AP-1 promoter was significantly increased with *Wdr62* transfection. Interestingly, the activity of the AP-1 promoter was synergistically activated by *Wdr62* transfection and RA treatment (Fig 4C). Moreover, RA induced JNK signaling activation was significantly decreased when endogenous *Wdr62* was knocked down with shRNA in F9 cells (Fig 4E±4G). We also found that the expression of *Stra8* could be induced by JNK1 and JNK2 overexpression in F9 cells (Fig 4D).

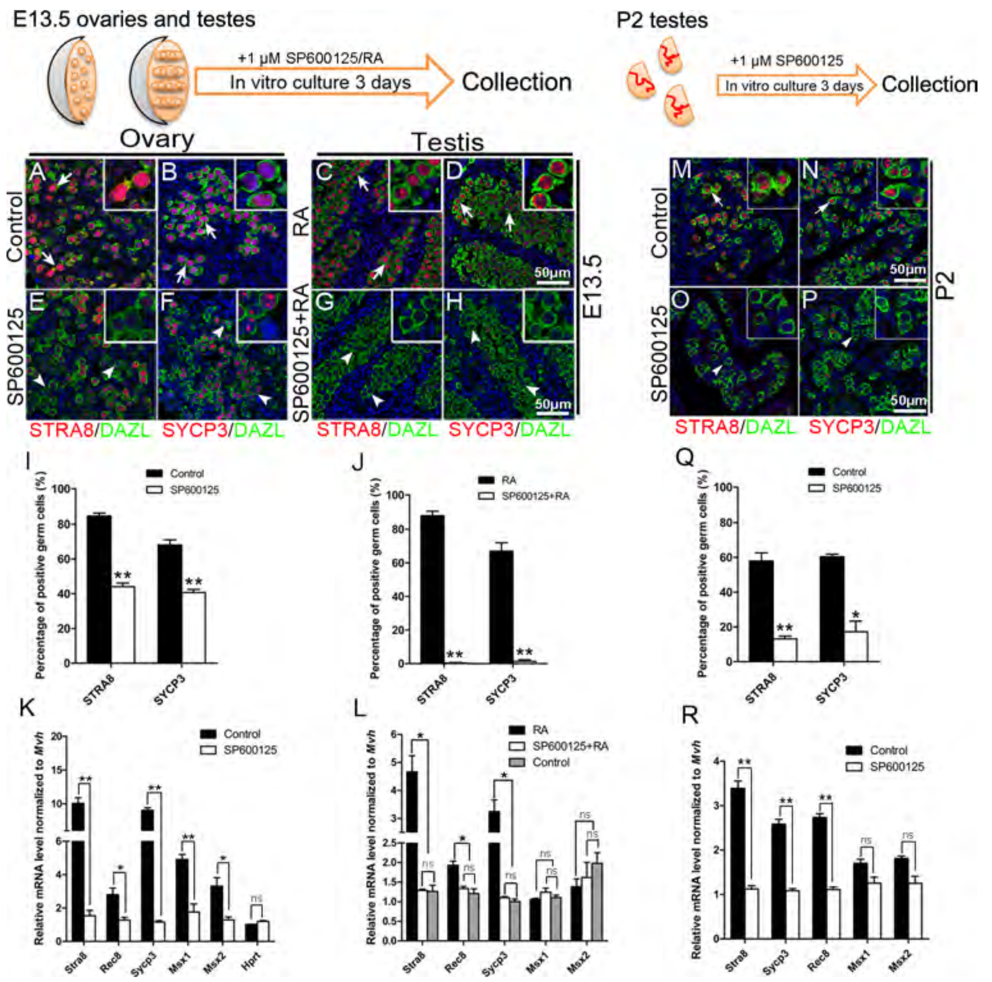

Fig 5. RA-induced meiosis-associated gene expression was attenuated by JNK inhibitor treatment. Control ovaries and testes at E13.5 and P2 were cultured in vitro and treated with 1 μM RA and/or 1 μM JNK inhibitor SP600125. The expression of meiotic genes was examined by immunofluorescence and real-time PCR. The number of (E) STRA8-positive germ cells (red, white arrowheads) in the ovaries was dramatically reduced when SP600125 was present in the culture medium compared with the (A) control group (red, white arrows). (F) The expression of SYCP3 in female germ cells was also dramatically reduced with SP600125 treatment, and meiosis was blocked during early leptotene stage (white arrowheads) compared with the (B) control group (red, white arrows). (C and D) STRA8- and SYCP3-positive germ cells (white arrows) were detected in the testes with RA treatment. (G and H) No STRA8- or SYCP3-positive germ cells (white arrowheads) were noted in the testes with combined RA and SP600125 treatment. (I and J) Quantitative analyses of STRA8- and SYCP3-positive germ cells in control and RA/SP600125-treated ovaries and testes. (K) The mRNA levels of meiotic genes were significantly decreased in SP600125-treated ovaries compared with control ovaries. (L) RA-induced meiotic gene expression in male germ cells was completely blocked by the JNK inhibitor SP600125 treatment. The number of STRA8- and SYCP3-positive germ cells in (O and P) the SP600125-treated testes was dramatically reduced compared with (M and N) the control group. (Q) Quantitative analyses of STRA8- and SYCP3-positive germ cells in control and SP600125-treated testes. (R) The mRNA level of meiotic genes was significantly decreased in SP600125-treated testes compared with control testes. Data are presented as the mean ± SEM. ns, $p > 0.05$, $^{*}p < 0.05$; and $^{**}p < 0.01$.

https://doi.org/10.1371/journal.pgen.1007463.g005

To further examine the functions of JNK signaling in meiotic gene expression, ovaries and testes from E13.5 control embryos and P2 testes were cultured in vitro and treated with RA and/or JNK inhibitor SP600125. Immunostaining results showed that STRA8 protein level was dramatically reduced (Fig 5E) and that the germ cells were blocked at early leptotene stage in the ovaries when treated with 1 μM SP600125 (Fig 5F). The percentage of STRA8- and SYCP3-positive germ cells in SP600125-treated ovaries was significantly reduced compared to

that of control group (Fig 5I). The mRNA levels of meiosis-related genes were also significantly reduced in SP600125-treated ovaries (Fig 5K). As expected, STRA8 and SYCP3 expression were detected in the testes after exogenous RA treatment (Fig 5C and 5D). However, no STRA8 and SYCP3 proteins were detected in the testes with combined RA and SP600125 treatment (Fig 5G, 5H and 5J). The mRNA levels of *Stra8*, *Rec8* and *Sycp3* were significantly reduced compared with that in RA-only treated testes (Fig 5L). In testes, after 3 days culture, the expression of STRA8 and SYCP3 proteins was dramatically reduced with SP600125 treatment (Fig 5O and 5P) compared with control group (Fig 5M and 5N). The results of quantitative analyses showed that the percentage of STRA8- and SYCP3-positive germ cells in SP600125-treated testes was evidently reduced compared with control group (Fig 5Q). The mRNA level of meiotic genes was also significantly decreased in SP600125-treated testes compared with that in control testes (Fig 5R). These results indicated that JNK signaling plays important roles in germ cell meiotic initiation, and demonstrated that the defect in germ cell development in *Wdr62* knockout mice was most likely mediated by JNK signaling.

To test this hypothesis, *Wdr62*$^{-/-}$; *CAJNK1*$^{\pm/flox}$; *Tnap-Cre* mice (referred to as rescued mice thereafter) were obtained. In this mouse model, *Wdr62* was completely inactivated and JNK1 was constitutively activated in germ cells from approximately E8.5. Strikingly, the number of germ cells was significantly increased in the ovaries from rescued mice at E13.5 compared with ovaries from *Wdr62*$^{-/-}$ mice (S12J Fig). Meiotic gene (STRA8, SYCP3 and γH2AX) expression was significantly increased in the germ cells from rescued mice at E13.5 (S12B, S12E, S12H and S12K Fig). A large number of developing follicles were observed in the ovaries of rescued mice 7 days after birth (Fig 6C and 6D). Because the defect of germ cell development in rescued mice was only partially recovered, it is very hard to get pregnant spontaneously. To test the functions of oocytes from rescued mice, superovulation experiment was performed. After super-ovulation and mating with wild-type males, fertilized oocytes were obtained from 8-week-old rescued females. Live pups were obtained from both control and rescued females (Fig 6H and 6I) after embryo transplantation, and the quantitative information was shown in S2 Table. These results indicated that the defect of germ cell development in *Wdr62*$^{-/-}$ mice was partially rescued by JNK1 overexpression. Based on these results, we concluded that JNK signaling pathway plays important roles in *Wdr62*-dependent RA-induced meiotic gene expression.

WDR62 missense mutation and frameshift-deletion were detected in patients with primary amenorrhea (PA)

The phenotype observed in *Wdr62*-deficient females correlate well with the pathological changes in PA patients, who exhibit many more severe defects in ovarian function compared with those with secondary amenorrhea in POI patients. We found two *WDR62* heterozygous mutations by the screening of the whole exome sequencing in two patients with primary amenorrhea, and then we verified these mutations through Sanger sequencing. As shown in Fig 7A, Sanger sequencing revealed two novel mutations in the *WDR62* gene in two patients. The missense mutation c.G1796A (p. C599Y) located on exon 14 and the frameshift-deletion c.3203_3206del (P.T1068fs) located on exon 26. Although the software recognizes the base "G" in the sequence diagram on the left, it can be seen from the sequence diagram that it is A and G heterozygous (green curve represents A base, and black curve represents G base). A mouse homolog was identified, indicating conservation of this protein. No other mutation associated with infertility was detected in POF916 patient. Two mutations of *BRCA2* and one mutation of *SPTB* were detected in POF1072 patient. The *BRCA2*-deficient mice fail to progress complete meiosis, whereas meiotic initiation is normal [23]. The *SPTB* mutation information regarding

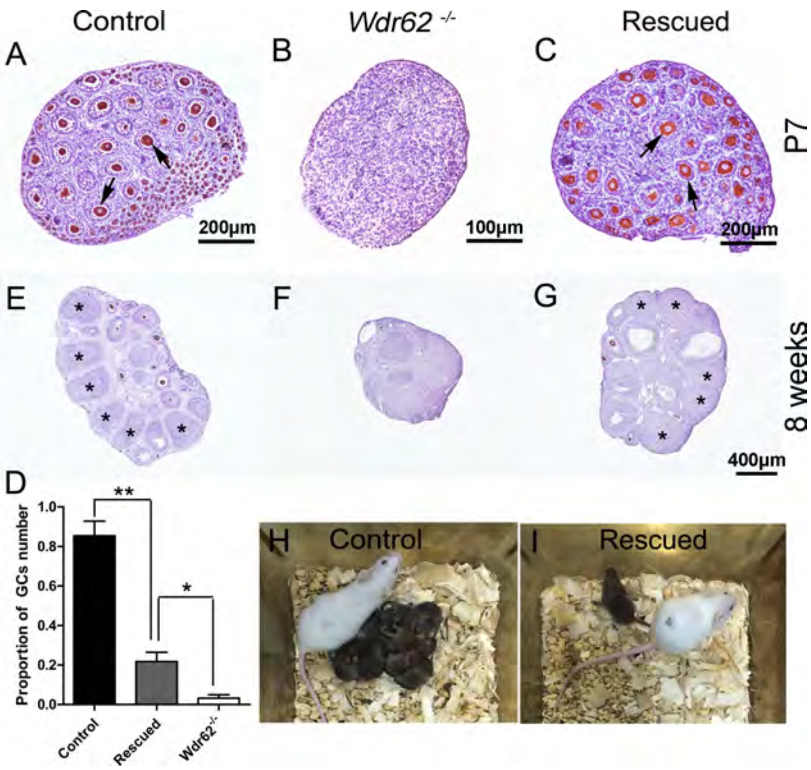

Fig 6. The defects of germ cell development in *Wdr62*$^{-/-}$ females were partially rescued by JNK1 overexpression. Numerous MVH-positive germ cells and growing follicles were observed in (A) control (arrows) and (C) rescued ovaries (arrows) at P7, but not in (B) *Wdr62*$^{-/-}$ ovaries. (D) Quantification of germ cell numbers in control, rescued and *Wdr62*$^{-/-}$ ovaries at P7. Many corpora lutea (CL) were observed in (E) 8-week control (asterisks) and (G) rescued ovaries (asterisks) primed with PMSG and hCG, but not in (F) ovaries from *Wdr62*$^{-/-}$ mice. Live pups were obtained from (H) control and (I) rescued *Wdr62*$^{-/-}$ females via superovulation and embryo transplantation. Data are presented as the mean ± SEM. ns, $p > 0.05$, $^{*}p < 0.05$; and $^{**}p < 0.01$.

https://doi.org/10.1371/journal.pgen.1007463.g006

infertility in mouse model can be found in Mouse Genome Informatics (http://www.informatics.jax.org). The detailed genetic profiles for these two patients were shown in supplementary material. Neither of the *WDR62* mutations was reported in either 1000 Genomes or dbSNP database. Further Sanger sequencing confirmed that both variants were absent in the 192 healthy controls (Fig 7A and 7B).

To test whether the normal function of WDR62 was affected by these mutations, a luciferase assay was performed with a *Stra8* promoter reporter vector and *Wdr62*-expressing vectors carrying a missense mutation (M1) or a frameshift-deletion (M2). The *Stra8* promoter was significantly activated by wild-type *WDR62* when RA was present, but it was not activated by mutant *WDR62* (M1 and M2). Additionally, *WDR62*-induced *Stra8* promoter activity was attenuated by co-transfection with mutant *WDR62* (M1 or M2) (Fig 7C). These results indicated that the mutations observed in human patients played a dominant-negative role in regulating *Stra8* expression and that mutation of *WDR62* is a potential etiology of POI in humans.

Discussion

Germ cell loss and infertility caused by meiotic defects have been reported previously [8, 24, 25]. In the *Wdr62*-deficient mouse model, germ cell loss was observed in both female and male mice, and the timing of germ cell loss in *Wdr62*-deficient mice was consistent with the developmental stage for meiotic initiation. The absence of meiosis-specific gene expression and

A

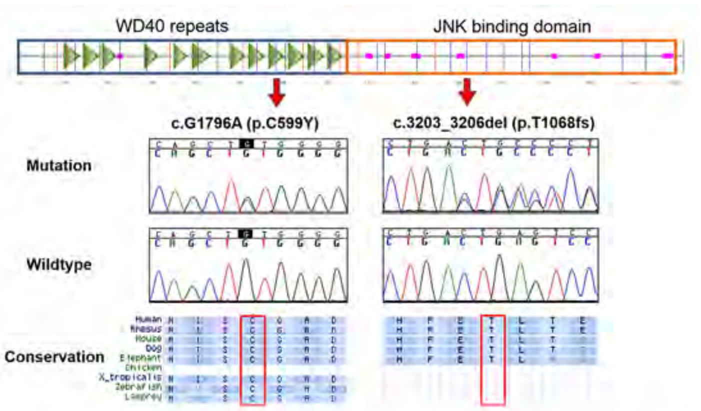

B

Patient No.	Mutation	Location	Sequence variation	Amino acid variation	Mutation rate n/N (%)	
					PA	Control
POF1072	Novel	Exon14	c.G1796A	p.C599Y	1/50(2)	0/192(0)
POF916	Novel	Exon26	c.3203_3206del	p.T1068fs	1/50(2)	0/192(0)

The table only refers to novel non-synonymous mutations and all mutations are heterozygous.
PA: primary amenorrhea.

C

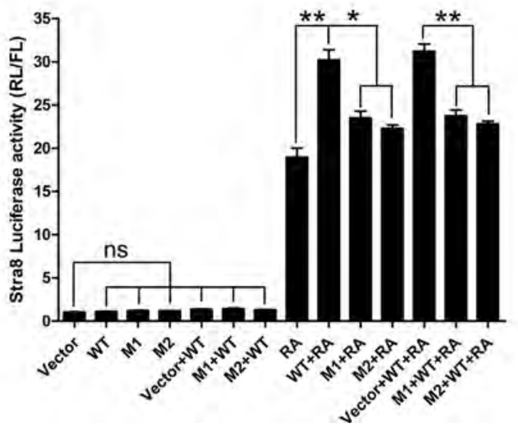

Fig 7. Mutations of *WDR62* gene were detected in patients with premature ovarian insufficiency (POI). (A) Genomic structure of *WDR62* and chromatograms of two mutations identified in POI patients by Sanger sequencing. Alignment of the coding strand of *WDR62* in nine eutherian mammals from Ensembl database. (B) The characteristics of *WDR62* novel mutations detected in the two patients. (C)The mutations of *WDR62* gene observed in POI patients play a dominant negative role in regulating *Stra8* expression. The *Stra8* promoter activity induced by wild-type *WDR62* (WT) and *WDR62* carrying the mutations detected in POI patients (M1 and M2) was analyzed by luciferase assay. The *Stra8* promoter was activated by WT when RA was present, but not by mutants. The *WDR62* induced *Stra8* promoter activity was attenuated by co-transfection of WT and mutant expression vectors. Data are presented as the mean ± SEM. ns, $p > 0.05$, $^{*}p < 0.05$; $^{**}p < 0.01$.

https://doi.org/10.1371/journal.pgen.1007463.g007

chromatin condensation in *Wdr62*-deficient germ cells further confirmed the defects in meiotic initiation after *Wdr62* inactivation. Although the expression of meiotic genes was absent, the expression of germ cell-specific genes (such as *Dazl* and *Mvh*) was not affected in *Wdr62*-deficient germ cells. Germ cells maintain pluripotency before meiotic initiation. Upon meiotic initiation, the pluripotency program is switched off and a set of genes is turned on to enable their differentiation. In *Wdr62*-deficient germ cells, the expression of core pluripotency genes (*Oct4*, *Nanog*, *Sox2* and *Stella*) was all significantly increased compared with the control germ cells. This phenomenon is also observed in *Stra8* and *Dazl* knock-out germ cells [5, 8],

indicating that the process of meiosis is blocked and that the germ cells are retained in an undifferentiated state.

Organ culture experiments showed that *Stra8* expression could not be induced by RA treatment in *Wdr62*-deficient germ cells. However, an in vitro study showed that *Stra8* mRNA level was significantly induced after RA treatment without *Wdr62* transfection. This discrepancy is most likely due to the endogenous *Wdr62* in F9 cells.

Stra8 expression was completely absent, whereas *Dazl* expression was not affected in *Wdr62*-deficient germ cells, suggesting that *Wdr62* is required for RA-induced *Stra8* expression, which is probably independent of *Dazl*. Based on these results, we concluded that *Wdr62* is required for germ cell meiotic initiation and that the germ cell loss in *Wdr62*-deficient mice is most likely a consequence of meiotic defects.

Although diverse cell types are exposed to RA during embryo development [26], meiotic initiation is limited to the germ line. Moreover, embryonic germ cells do not respond to RA induction until they migrate into the developing gonad, suggesting that RA alone is not sufficient to induce the temporal and cell-type-specific meiotic initiation. Other intrinsic factors are required for this process. *Dazl* has been demonstrated to play an essential role for meiotic initiation as an intrinsic factor [7]. Inactivation of *Dazl* leads to a lack of *Stra8* expression and meiotic initiation defects [7]. However, whether other signaling pathways are also involved in meiotic initiation is unknown. JNK signaling pathway has been reported to play important roles in multiple organ development, and inactivation of JNK1 and JNK2 causes embryonic lethality in mouse model [27]. In this study, we found that JNK signaling was induced by RA treatment and *Wdr62* was required for RA-induced JNK signaling activation in germ cells. The function of JNK signaling in meiosis has been further confirmed by organ culture experiments. We found that RA-induced meiotic gene expression in germ cells was abolished by treatment with a JNK inhibitor. Most importantly, the defect in germ cell development from *Wdr62*-deficient mice could be partially rescued by overexpression of constitutively activated JNK1. Our study demonstrated for the first that JNK signaling is involved in germ cell meiotic initiation. However, the underlying mechanism is still unclear and need further investigation.

POI is highly heterogeneous, with no single underlying dominant gene deficiency. More than 80 candidate genes have been identified, with approximately 25% demonstrating causative function. Elucidating the etiology and molecular basis of POI is of paramount importance for understanding ovarian physiology. The two mutations in the *WDR62* gene identified in POI patients with a dominant-negative role provide clinical evidence for the role of *WDR62* in folliculogenesis.

The gene trap mouse model of *Wdr62* has been reported previously. In that mouse model, a β-geo reporter was inserted in the intronic region between exon 14 and 15, which caused a down-regulation of *Wdr62* expression [19]. The homozygous mutant mice exhibited growth retardation and reduced brain size with spindle instability and mitotic arrest in neural progenitor and MEF cells. In our mouse model, exon 2 was deleted which caused a frame-shift of the *Wdr62* gene. Surprisingly, we found that *Wdr62*$^{-/-}$ mice were born at a normal Mendelian ratio, and no obvious developmental defects were observed. This discrepancy between the two mouse models is probably caused by the different strategies used for generating the mouse models.

In summary, this study demonstrated that *Wdr62* is required as a pivotal permissive factor for meiotic initiation in germ cells via activating JNK signaling and that mutation of *WDR62* is one of the potential etiologies of POI in humans. The results from this study reveal a novel mechanism for regulating meiotic initiation, which will allow us to better understand the regulation of meiotic initiation.

Methods

Ethics statement

All animal experimental procedures involved were performed in accordance with protocols approved by the Institutional Animal Care and Use Committee (IACUC) of the Institute of Zoology, CAS (AEI-09-02-2014). This human research study was approved by the Institutional Review Board of Reproductive Medicine of Shandong University ([2012] IRB No.18). Written informed consent was obtained from all subjects.

Generation of *Wdr62* knock-out mouse strain

A $Wdr62^{+/flox}$ mouse model was generated based on methods reported previously [28]. In brief, the $Wdr62^{flox}$ allele was generated by inserting two loxp sites at both sides of exon 2 via homologous recombination. The $Wdr62^{+/-}$ mouse strain was obtained by crossing with *ZP3-Cre* transgenic mice. In *ZP3-Cre* mice, Cre recombinase is specifically expressed in oocytes [29]. In this mouse model, exon 2 was deleted, which caused a frame shift. The strategy for gene targeting in ES cells and genotyping was shown in S2 Fig.

Mice

All mice were maintained on a C57BL/6;129/SvEv mixed background. $Wdr62^{-/flox}$; *Tnap-Cre* mice were obtained by crossing $Wdr62^{+/-}$; *Tnap-Cre* males with $Wdr62^{flox/flox}$ females. The rescued mice ($Wdr62^{-/-}$; $CAJNK1^{+/flox}$; *Tnap-Cre*) were obtained by crossing $Wdr62^{+/-}$; *Tnap-Cre* males with $Wdr62^{+/-}$; $CAJNK1^{flox/flox}$ females. DNA isolated from tail biopsies and fetal tissues was used for genotyping as described previously [30, 31].

Tissue collection and histological analysis

Gonads dissected from knock-out and control mice immediately after euthanasia, were fixed in 4% paraformaldehyde for up to 24 hours, stored in 70% ethanol, and embedded in paraffin. Five-micrometer-thick sections were cut and mounted on glass slides. After deparaffinization, sections were processed for immunohistochemistry and immunofluorescence analysis.

Immunohistochemistry, immunofluorescence, TUNEL assay and quantification analyses

IHC and IF procedures were performed as described previously [30]. Antibodies were diluted as follows: MVH (1:500, Abcam, ab13840), WDR62 (1:400, Bethyl, A301-560A), DAZL (1:100, AbD Serotec, MCA2336), STRA8 (1:200, Abcam, ab49405), SYCP3 (1:200, Abcam, ab15093), γH2AX (1:400, Millipore, 05±636), Ki67 (1:200, Abcam, ab15580). After staining, the sections were examined with a Nikon microscopy, and images were captured with a Nikon DS-Ri1 CCD camera. The IF sections were examined using a confocal laser scanning microscope (Carl Zeiss Inc., Thornwood, NY). TUNEL assay was performed using the DeadEnd Fluorometric TUNEL System (Promega, G3250).

For quantitative analyses, more than three biological replicates from the control and experimental groups were performed. Paraffin-embedded ovaries and testes were serially sectioned and at least three sections apart were stained for observation. Within the group, at least three cross sections from each animal were examined. Around 1000 germ cells were counted in each group. The quantification of germ cells number (GCs number) was normalized to the control group. We considered the number of germ cells in the control group as 1, then the number of

germ cells in other groups were quantified relative to the number of germ cells in the control group.

Germ cell isolation

Germ cells were isolated from E13.5 genital ridges using SSEA-1 antibody as previously described [32]. Briefly, the digested female gonads were pooled and transferred to an Eppendorf tube containing 0.25% trypsin-0.02% EDTA for 5 min at 37ÊC.After digestion, a single-cell suspension was obtained by repeated pipetting. Then, 20 μL of SSEA-1 microbeads (Miltenyi) were added to the single-cell suspension and incubated for 15 min at 4ÊC.A magnetic separation was used to collect the magnetically labeled germ cells by applying the cell suspension onto a column (Miltenyi) placed in a MiniMACS separation unit (Miltenyi).

Nucleic acid isolation and quantitative reverse transcription PCR (RT-PCR)

Total RNA was extracted from MACS sorted cells, cultured F9 cells or gonads using a Qiagen RNeasy kit in accordance with the manufacturer's instructions. Two micrograms of total RNA was used to synthesize first-strand cDNA. To quantify gene expression, a SYBR Green real-time PCR assay was performed with the isolated RNA. All gene expression analyses shown in Fig 2, Fig 4 and S2 Fig were quantified relative to *Gapdh* expression, and *Mvh* was used as an endogenous control for gene expression analysis in the other figures (Fig 3, Fig 5 and S1 Fig). The relative concentration of the candidate genes was calculated using the formula $2^{-\Delta\Delta CT}$ as described in the SYBR Green user manual. The primers used were listed in supplementary S1 Table.

Western blotting

Tissue and cell were extracted in cold RIPA buffer (25 mM Tris-HCl, pH 7.6, 150 mM NaCl, 1%NP-40, 1% sodium deoxycholate, and 0.1% sodium dodecyl sulfate), which supplemented with 1 mM phenylmethylsulfonyl fluoride and a protease inhibitor cocktail (Roche, Indianapolis, IN, USA). The protein lysates were resolved by SDS±polyacrylamide gel electrophoresis (PAGE), transferred onto a nitrocellulose membrane and probed with the primary antibodies. The images were captured with the ODYSSEY Sa Infrared Imaging System (LI-COR Biosciences, Lincoln, NE, USA). Antibodies were diluted as follows: WDR62 (1:1000, Abcam, ab154044), Phospho-SAPK/JNK (Thr183/Tyr185) (1:1000, CST, 9251).

Plasmid construction

The promoter region of the *Stra8* and *Ap-1* genes was amplified by PCR using pfu DNA polymerase (NEB) as reported previously [33±35]. The amplified fragments were cloned into pGL3-basic plasmids. The *Wdr62* expression vector was amplified by PCR using pfu DNA polymerase (NEB) and cloned into a pcDNA3.1-HA vector. The *WDR62* mutation vectors carrying p.C599Y and p.T1068fs mutations were constructed by point mutagenesis using KOD--Plus-Neo polymerase (TOYOBO). The primers used for cloning were listed in S1 Table.

Plasmid transfection and luciferase assay

Cells of the mouse embryonal carcinoma F9 cell line were plated in 24-well plates and transfected with plasmids using Lipofectamine 3000 (Invitrogen) according to the manufacturer's instructions. Forty-eight hours after transfection, the cells were lysed, and whole-cell extracts from triplicate wells were analyzed. pRL-TK (Promega) containing the Renilla luciferase gene

was used as an internal control. The luciferase activity was measured with a luminometer (Promega). The results were normalized against Renilla luciferase activity.

Organ culture

Agarose gel stands (1.5% w/v, placed in 24-well plates) were pre-incubated with culture medium for more than 24 hours. The gonads with mesonephroi were dissected from control and *Wdr62*-deficient E13.5 embryos and P2 testes were dissected, then placed them on agarose stands. The gonads were cultured in DMEM/F12 containing 10% FBS at 37ÊCand 5% CO_2. Then, 1 μM RA and/or 1 μM SP600125 were added to the culture medium. The cultured gonads were collected 72 hours later, and the gene expression was analyzed by real-time PCR and IF analysis.

Superovulation and embryo transplantation

Each female mouse at 8-week-old was injected with 7.5 IU of PMSG followed by 7.5 IU of hCG 48 hours to promote ovulation. The fertilized oocytes were collected from the ampulla of the oviduct of superovulated female mice after mating with male mice. The fertilized oocytes were transferred to the oviduct of surrogate mother in ICR background.

Whole exome sequencing in 50 patients with POI

A total of 50 unrelated Han Chinese POI women with primary amenorrhea (PA) were recruited between April 2003 and Nov 2016 from the Center for Reproductive Medicine, Shandong Provincial Hospital Affiliated to Shandong University. Among patients with POI, women with PA exhibit much more severe defects in ovarian function compared with those with secondary amenorrhea. The inclusion criteria were absence of menstruation by the age of 16 with a serum follicle stimulating hormone (FSH) level >40 IU/L, measured on two occasions at least one month apart. Patients with known chromosomal abnormalities, previous chemo-/radiotherapy or ovarian surgery, autoimmune disorders, or somatic anomalies (particularly any reported as associated with syndromic POI) were excluded. In addition, 192 age-matched women with regular menses and a normal FSH level were enrolled as controls. Clinical characteristics of sporadic patients with PA and controls were shown in S3 Table.

We preformed whole exome sequencing (WES) in the 50 patients with PA. Genomic DNA was extracted from peripheral blood with QIAamp DNA Blood kit (QIAGEN, Hilden, Germany) according to standard protocols. Whole exome capture was carried out with SureSelect Target Enrichment System for Illumina Paired-End Sequencing Library (Agilent Technologies, Santa Clara, CA). DNA sequencing were performed on the Illumina platform (Illumina Hiseq, San Diego, CA). Reads were mapped to the hg19 reference genome with Burrows-Wheeler Alignment (BWA), and variants were called and annotated using ANNOVAR. Protein-coding variants were checked against established databases (1000 Genomes Project and dbSNP, version138). Novel nonsynonymous variants, which were predicted to below 0.05 on SIFT website and above 0.95 on PolyPhen-2 website, were confirmed through Sanger sequencing on two occasions. Nomenclature of variants identified was established according to Human Genome Variation Society (HGVS, www.hgvs.org/mutnomen).

Sanger sequencing

Genomic DNA was extracted from peripheral blood according to standard protocols. The target fragments of *WDR62* (NM_001083961) were amplified by PCR using primers listed in S1 Table. The PCR products was purified, labeled by BigDye (Terminatorv3.1 Cycle Sequencing

Kits, Applied Biosystems), and sequenced by ABI 37306l DNA Analyzer (Applied Biosystems, Foster City, CA). The two variants were confirmed by two independent PCR runs and sequenced in both forward and reverse strands.

Statistical analysis

All experiments were repeated at least three times and at least three individual animals of each genotype were performed. The quantitative results were presented as the mean±SEM. The data were analyzed with Student's t-test and one-way ANOVA.

Supporting information

S1 Fig. *Wdr62* expression in male and female germ cells. *Wdr62* expression in the ovaries and testes was examined by immunohistochemistry and real-time PCR. WDR62 protein was abundantly expressed in (A) female and (B) male germ cells from control mice. No WDR62 protein was detected in germ cells from *Wdr62*-defecient (D) ovaries and (E) testes at E13.5. (C) Brain IHC staining as a positive control. (F) The mRNA level of *Wdr62* in ovaries and testes at E13.5 and E15.5. (G) *Wdr62* mRNA levels were gradually increased from E11.5 to E13.5 and dramatically decreased at E14.5 and E16.5 in female gonads. (H) In testes, *Wdr62* mRNA levels were significantly increased from P1 to P7 and dramatically decreased at P10. Data are presented as the mean ± SEM.
(TIF)

S2 Fig. Generation of *Wdr62* knock-out mouse strains. (A) Gene-targeting strategy for generating the $Wdr62^{flox}$ allele. (B) Upper panel: PCR analysis using primers F2/R2. Middle panel: PCR analysis using primers F3/R3. Lower panel: PCR analysis using primers F1/R1. ES clone A2 is the positive clone, containing both LoxP sites and Neo, and was homologously recombined into genomic DNA. (C) Genotyping of mice. +, WT; Fl, targeted; -, deleted. (D) Real-time PCR analysis showed the efficiency of *Wdr62* knock-out in the female germ cells at E13.5. Data are presented as the mean ± SEM. ns, $p > 0.05$; $^{*}p < 0.05$; $^{**}p < 0.01$.
(TIF)

S3 Fig. Germ cell loss was noted in *Wdr62*-deficient ovaries at E13.5. Germ cells were labeled with anti-MVH antibody. The number of germ cells in the (B) $Wdr62^{-/-}$ ovaries (black arrowheads) was not changed at E12.5 compared with (A) the control ovaries (black arrows). The number of MVH-positive germ cells was significantly reduced in $Wdr62^{-/-}$ ovaries at (D) E13.5 and (F) E15.5 compared with (C and E) control ovaries. (G) Numerous germ cells (black arrows) were observed in control ovaries at P1, whereas (H) very few MVH-positive germ cells (black arrowheads) were noted in *Wdr62*-deficient ovaries at P1. (I) Quantification of germ cell numbers in control and $Wdr62^{-/-}$ ovaries at different developmental stages. Data are presented as the mean ± SEM. ns, $p > 0.05$; $^{*}p < 0.05$; $^{**}p < 0.01$.
(TIF)

S4 Fig. The gross images and weights of testes. (A-C) The size of $Wdr62^{-/-}$ testes was comparable to the control testes at P1, and smaller than controls at P5 and P10. (D) The weight of $Wdr62^{-/-}$ testes was significantly decreased at P5 and P10. Data are presented as the mean ± SEM. ns, $p > 0.05$; $^{*}p < 0.05$; $^{**}p < 0.01$.
(TIF)

S5 Fig. Germ cell loss was observed in *Wdr62*-deficient testis at P5. Germ cells were labeled with anti-MVH antibody. (B and D) The number of germ cells in $Wdr62^{-/-}$ testes was not changed at E15.5 and P1 (black arrowheads), respectively, compared with (A and C) the

control testes (black arrows). (F) The germ cell loss in *Wdr62*$^{-/-}$ testes (black arrowheads) was noted at P5, and (H) very few germ cells were observed in *Wdr62*-deficient testes at P10 (black arrowheads). (I) Quantification of germ cell numbers in control and *Wdr62*$^{-/-}$ testes at different developmental stages. Data are presented as the mean ± SEM. ns, $p > 0.05$; $^{*}p < 0.05$; $^{**}p < 0.01$.
(TIF)

S6 Fig. Inactivation of *Wdr62* specifically in germ cells led to germ cell loss. To examine the functions of *Wdr62* was specifically in germ cells, *Wdr62*$^{+/-}$*; Tnap-Cre* males were crossed with *Wdr62*$^{flox/flox}$ females to obtain *Wdr62*$^{-/flox}$*; Tnap-Cre* offspring, in which Cre is activated in germ cells of ovaries and testes at approximately 8.5 dpc at embryo stage. It is shown that few germ cells were survived in the (B, black arrowheads) ovaries and (D, black arrowheads) testes of *Wdr62*$^{-/flox}$*; Tnap-Cre* mice compared with that of (A and C, black arrows) control mice at P7.
(TIF)

S7 Fig. No defect of germ cell development was observed in *Wdr62*$^{-/\mathrm{flox}}$*; Amh-Cre* mice. Compared with (A, B and C) control mice, the germ cell development in (D, E and F) *Wdr62*$^{-/\mathrm{flox}}$*; Amh-Cre* mice was not affected. (F) A large number of mature sperm were observed in the epididymis of *Wdr62*$^{-/\mathrm{flox}}$*; Amh-Cre* mice.
(TIF)

S8 Fig. The expression of meiosis-related genes was dramatically reduced in germ cells from *Wdr62*-deficient testes. The expression of meiosis-related genes in germ cells was examined by immunofluorescence. In control testes, (A, red) STRA8 was detected in most germ cells (green, white arrows) at P3. (B, red, white arrows) SYCP3 and (C, red, white arrows) λH2AX were detected in control germ cells (green, white arrows) at P5. (D, E and F) None of these proteins was expressed in germ cells (white arrowheads) from *Wdr62*-deficient testes at these stages. (G) Quantitative analyses of meiotic germ cells from control and *Wdr62*-deficient testes at P3 and P5. Data are presented as the mean ± SEM. ns, $p > 0.05$; $^{*}p < 0.05$; $^{**}p < 0.01$.
(TIF)

S9 Fig. No meiotic chromosome condensation was observed in germ cells from *Wdr62*-deficient ovaries at E13.5. Hematoxylin and eosin staining of ovarian sections from the control and *Wdr62*-deficient littermate embryos. The germ cells displayed a morphology with patches of condensed chromatin at the periphery of the nucleus in both (A, white arrows) control and (C, white arrowheads) *Wdr62*–/– ovaries at E12.5. (B, white arrows) By E13.5, the nuclei in many control germ cells showed the thread±like chromosome condensation that represents the pre-leptotene stage, (D, white arrowheads) whereas the nuclei from *Wdr62*-deficient germ cells retained the morphology of the mitotic stage as observed at E12.5.
(TIF)

S10 Fig. Ki67 immunostaining and TUNEL assay. The proliferation and apoptosis of germ cells was examined by Ki67 staining and TUNEL assay. Ki67 signal (red) was detected in most germ cells (green, white arrows) at E11.5 and E12.5 from (A and B) control and (E and F) *Wdr62*-deficient ovaries. (C and D) Very few Ki67-positive germ cells were detected in control ovaries at E13.5 and E15.5, (G and H) whereas numerous germ cells in *Wdr62*-deficient ovaries were retained Ki67-positive at E13.5 and E15.5. (I) The quantification analyses of Ki67-positive germ cells in control and *Wdr62*$^{-/-}$ ovaries at different developmental stages. (J-Q) Representative images of TUNEL assay of control and *Wdr62*$^{-/-}$ ovaries. (R) Quantitative analyses of TUNEL-positive germ cells in control and *Wdr62*$^{-/-}$ ovaries. Data are presented as the

mean ± SEM. ns, $p > 0.05$; $^{*}p < 0.05$; $^{**}p < 0.01$.
(TIF)

S11 Fig. The immunostaining of phosphorylated JNK protein. The expression of p-JNK in germ cells at E13.5 was examined by immunofluorescence. In (A and B) control mice, p-JNK was detected in a small portion of germ cells (green, white arrows), whereas very few p-JNK positive germ cell (green, white arrowheads) was noted in (C and D) *Wdr62*-deficient mice.
(TIF)

S12 Fig. The deficiency of meiotic gene expression was rescued by JNK1 overexpression in germ cells. The expression of meiotic genes was examined by immunofluorescence. (A and B, white arrows) STRA8, (D and E, white arrows) SYCP3 and (G and H, white arrows) γH2AX were detected in germ cells from (A, D and G) control and (B, E and H) rescued ovaries, but not in the (C, F and I) *Wdr62*$^{-/-}$ ovaries. The number of germ cells was also significantly increased in the (B, E, and H) rescued ovaries compared with the (C, F and I) *Wdr62*$^{-/-}$ ovaries. (J) Quantification of germ cell numbers in control, rescued and *Wdr62*-deficient ovaries at E13.5. (K) Quantitative analyses of meiotic germ cells in control, rescued and *Wdr62*-deficient ovaries at E13.5. Data are presented as the mean ± SEM. ns, $p > 0.05$; $^{*}p < 0.05$; $^{**}p < 0.01$.
(TIF)

S1 Table. Primers used for real-time PCR and mutagenesis analysis.
(DOCX)

S2 Table. Superovulation and embryo transplantation.
(DOCX)

S3 Table. Clinical features of sporadic patients with PA and matched controls.
(DOCX)

Author Contributions

Formal analysis: Yang Zhou, Min Chen.

Funding acquisition: Fei Gao.

Investigation: Yang Zhou, Binyang Xu.

Methodology: Yang Zhou, Yan Qin, Yingying Qin, Ting Guo, Hanni Ke, Lianjun Zhang, Feng Han, Yaqiong Li, Min Chen, Yaqing Wang, Zhiheng Xu, Zi-Jiang Chen.

Project administration: Fei Gao.

Resources: Yingying Qin, Axel Behrens, Yaqing Wang, Zhiheng Xu, Zi-Jiang Chen.

Writing ± original draft: Yang Zhou.

Writing ± review & editing: Yang Zhou, Fei Gao.

References

1. Bowles J, Knight D, Smith C, Wilhelm D, Richman J, Mamiya S, et al. Retinoid signaling determines germ cell fate in mice. Science. 2006; 312(5773):596±600. https://doi.org/10.1126/science.1125691 PMID: 16574820
2. Koubova J, Menke DB, Zhou Q, Capel B, Griswold MD, Page DC. Retinoic acid regulates sex-specific timing of meiotic initiation in mice. Proceedings of the National Academy of Sciences of the United States of America. 2006; 103(8):2474±9. https://doi.org/10.1073/pnas.0510813103 PMID: 16461896

3. Miyamoto T, Sengoku K, Takuma N, Hasuike S, Hayashi H, Yamauchi T, et al. Isolation and expression analysis of the testis-specific gene, STRA8, stimulated by retinoic acid gene 8. Journal of assisted reproduction and genetics. 2002; 19(11):531±5. https://doi.org/10.1023/A:1020963919596 PMID: 12489526
4. Anderson EL, Baltus AE, Roepers-Gajadien HL, Hassold TJ, de Rooij DG, van Pelt AM, et al. Stra8 and its inducer, retinoic acid, regulate meiotic initiation in both spermatogenesis and oogenesis in mice. Proceedings of the National Academy of Sciences of the United States of America. 2008; 105(39):14976±80. https://doi.org/10.1073/pnas.0807297105 PMID: 18799751
5. Baltus AE, Menke DB, Hu YC, Goodheart ML, Carpenter AE, de Rooij DG, et al. In germ cells of mouse embryonic ovaries, the decision to enter meiosis precedes premeiotic DNA replication. Nature genetics. 2006; 38(12):1430±4. https://doi.org/10.1038/ng1919 PMID: 17115059
6. Mark M, Jacobs H, Oulad-Abdelghani M, Dennefeld C, Feret B, Vernet N, et al. STRA8-deficient spermatocytes initiate, but fail to complete, meiosis and undergo premature chromosome condensation. Journal of cell science. 2008; 121(Pt 19):3233±42. https://doi.org/10.1242/jcs.035071 PMID: 18799790
7. Lin Y, Gill ME, Koubova J, Page DC. Germ cell-intrinsic and -extrinsic factors govern meiotic initiation in mouse embryos. Science. 2008; 322(5908):1685±7. https://doi.org/10.1126/science.1166340 PMID: 19074348
8. Gill ME, Hu YC, Lin Y, Page DC. Licensing of gametogenesis, dependent on RNA binding protein DAZL, as a gateway to sexual differentiation of fetal germ cells. Proceedings of the National Academy of Sciences of the United States of America. 2011; 108(18):7443±8. https://doi.org/10.1073/pnas.1104501108 PMID: 21504946
9. Haller-Kikkatalo K, Uibo R, Kurg A, Salumets A. The prevalence and phenotypic characteristics of spontaneous premature ovarian failure: a general population registry-based study. Human reproduction. 2015; 30(5):1229±38. https://doi.org/10.1093/humrep/dev021 PMID: 25712230
10. Qin Y, Choi Y, Zhao H, Simpson JL, Chen ZJ, Rajkovic A. NOBOX homeobox mutation causes premature ovarian failure. American journal of human genetics. 2007; 81(3):576±81. https://doi.org/10.1086/519496 PMID: 17701902
11. Bouilly J, Bachelot A, Broutin I, Touraine P, Binart N. Novel NOBOX loss-of-function mutations account for 6.2% of cases in a large primary ovarian insufficiency cohort. Human mutation. 2011; 32(10):1108±13. https://doi.org/10.1002/humu.21543 PMID: 21837770
12. Zhao H, Chen ZJ, Qin Y, Shi Y, Wang S, Choi Y, et al. Transcription factor FIGLA is mutated in patients with premature ovarian failure. American journal of human genetics. 2008; 82(6):1342±8. https://doi.org/10.1016/j.ajhg.2008.04.018 PMID: 18499083
13. Rossetti R, Di Pasquale E, Marozzi A, Bione S, Toniolo D, Grammatico P, et al. BMP15 mutations associated with primary ovarian insufficiency cause a defective production of bioactive protein. Human mutation. 2009; 30(5):804±10. https://doi.org/10.1002/humu.20961 PMID: 19263482
14. Chand AL, Ponnampalam AP, Harris SE, Winship IM, Shelling AN. Mutational analysis of BMP15 and GDF9 as candidate genes for premature ovarian failure. Fertility and sterility. 2006; 86(4):1009±12. https://doi.org/10.1016/j.fertnstert.2006.02.107 PMID: 17027369
15. Nelson LM. Clinical practice. Primary ovarian insufficiency. The New England journal of medicine. 2009; 360(6):606±14. https://doi.org/10.1056/NEJMcp0808697 PMID: 19196677
16. Wasserman T, Katsenelson K, Daniliuc S, Hasin T, Choder M, Aronheim A. A novel c-Jun N-terminal kinase (JNK)-binding protein WDR62 is recruited to stress granules and mediates a nonclassical JNK activation. Molecular biology of the cell. 2010; 21(1):117±30. https://doi.org/10.1091/mbc.E09-06-0512 PMID: 19910486
17. Nicholas AK, Khurshid M, Desir J, Carvalho OP, Cox JJ, Thornton G, et al. WDR62 is associated with the spindle pole and is mutated in human microcephaly. Nature genetics. 2010; 42(11):1010±4. https://doi.org/10.1038/ng.682 PMID: 20890279
18. Xu D, Zhang F, Wang Y, Sun Y, Xu Z. Microcephaly-associated protein WDR62 regulates neurogenesis through JNK1 in the developing neocortex. Cell reports. 2014; 6(1):104±16. https://doi.org/10.1016/j.celrep.2013.12.016 PMID: 24388750
19. Chen JF, Zhang Y, Wilde J, Hansen KC, Lai F, Niswander L. Microcephaly disease gene Wdr62 regulates mitotic progression of embryonic neural stem cells and brain size. Nature communications. 2014; 5:3885. https://doi.org/10.1038/ncomms4885 PMID: 24875059
20. Jayaraman D, Kodani A, Gonzalez D, Mancias J, Mochida G, Vagnoni C, et al. Microcephaly Proteins Wdr62 and Aspm Define a Mother Centriole Complex Regulating Centriole Biogenesis, Apical Complex, and Cell Fate. Neuron. 2016.
21. Lomeli H, Ramos-Mejia V, Gertsenstein M, Lobe CG, Nagy A. Targeted insertion of Cre recombinase into the TNAP gene: excision in primordial germ cells. Genesis. 2000; 26(2):116±7. PMID: 10686602

22. Cohen-Katsenelson K, Wasserman T, Khateb S, Whitmarsh AJ, Aronheim A. Docking interactions of the JNK scaffold protein WDR62. The Biochemical journal. 2011; 439(3):381±90. https://doi.org/10.1042/BJ20110284 PMID: 21749326

23. Sharan SK, Pyle A, Coppola V, Babus J, Swaminathan S, Benedict J, et al. BRCA2 deficiency in mice leads to meiotic impairment and infertility. Development. 2004; 131(1):131±42. https://doi.org/10.1242/dev.00888 PMID: 14660434

24. Le Bouffant R, Souquet B, Duval N, Duquenne C, Herve R, Frydman N, et al. Msx1 and Msx2 promote meiosis initiation. Development. 2011; 138(24):5393±402. https://doi.org/10.1242/dev.068452 PMID: 22071108

25. Krentz AD, Murphy MW, Sarver AL, Griswold MD, Bardwell VJ, Zarkower D. DMRT1 promotes oogenesis by transcriptional activation of Stra8 in the mammalian fetal ovary. Developmental biology. 2011; 356(1):63±70. https://doi.org/10.1016/j.ydbio.2011.05.658 PMID: 21621532

26. Mark M, Ghyselinck NB, Chambon P. Function of retinoid nuclear receptors: lessons from genetic and pharmacological dissections of the retinoic acid signaling pathway during mouse embryogenesis. Annual review of pharmacology and toxicology. 2006; 46:451±480. https://doi.org/10.1146/annurev.pharmtox.46.120604.141156 PMID: 16402912

27. Sabapathy K, Jochum W, Hochedlinger K, Chang L, Karin M, Wagner EF. Defective neural tube morphogenesis and altered apoptosis in the absence of both JNK1 and JNK2. Mechanisms of development. 1999; 89(1±2):115±24. PMID: 10559486

28. Liu P, Jenkins NA, Copeland NG. A highly efficient recombineering-based method for generating conditional knockout mutations. Genome research. 2003; 13(3):476±84. https://doi.org/10.1101/gr.749203 PMID: 12618378

29. Lewandoski M, Wassarman KM, Martin GR. Zp3-cre, a transgenic mouse line for the activation or inactivation of loxP-flanked target genes specifically in the female germ line. Current biology. 1997; 7(2):148±51. PMID: 9016703

30. Gao F, Maiti S, Alam N, Zhang Z, Deng JM, Behringer RR, et al. The Wilms tumor gene, Wt1, is required for Sox9 expression and maintenance of tubular architecture in the developing testis. Proceedings of the National Academy of Sciences of the United States of America. 2006; 103(32):11987±92. https://doi.org/10.1073/pnas.0600994103 PMID: 16877546

31. Harada N, Tamai Y, Ishikawa T, Sauer B, Takaku K, Oshima M, et al. Intestinal polyposis in mice with a dominant stable mutation of the beta-catenin gene. The EMBO journal. 1999; 18(21):5931±42. https://doi.org/10.1093/emboj/18.21.5931 PMID: 10545105

32. Bowles J, Feng CW, Spiller C, Davidson TL, Jackson A, Koopman P. FGF9 suppresses meiosis and promotes male germ cell fate in mice. Dev Cell. 2010; 19(3):440±9. https://doi.org/10.1016/j.devcel.2010.08.010 PMID: 20833365

33. Giuili G, Tomljenovic A, Labrecque N, Oulad-Abdelghani M, Rassoulzadegan M, Cuzin F. Murine spermatogonial stem cells: targeted transgene expression and purification in an active state. EMBO reports. 2002; 3(8):753±9. https://doi.org/10.1093/embo-reports/kvf149 PMID: 12151334

34. Zhou H, Zarubin T, Ji Z, Min Z, Zhu W, Downey JS, et al. Frequency and distribution of AP-1 sites in the human genome. DNA research: an international journal for rapid publication of reports on genes and genomes. 2005; 12(2):139±50.

35. Weber WM, Hunsaker LA, Gonzales AM, Heynekamp JJ, Orlando RA, Deck LM, et al. TPA-induced up-regulation of activator protein-1 can be inhibited or enhanced by analogs of the natural product curcumin. Biochemical pharmacology. 2006; 72(8):928±40. https://doi.org/10.1016/j.bcp.2006.07.007 PMID: 16934760
